# Precise analyses of photoelectrochemical reactions on particulate Zn_0.25_Cd_0.75_Se photoanodes in nonaqueous electrolytes using Ru bipyridyl complexes as a probe†

**DOI:** 10.1039/d4sc00511b

**Published:** 2024-04-16

**Authors:** Yosuke Kageshima, Hiroto Takano, Mika Nishizawa, Fumiaki Takagi, Hiromu Kumagai, Katsuya Teshima, Kazunari Domen, Hiromasa Nishikiori

**Affiliations:** a Department of Materials Chemistry, Faculty of Engineering, Shinshu University 4-17-1 Wakasato Nagano 380-8553 Japan kage_ysk@shinshu-u.ac.jp nishiki@shinshu-u.ac.jp; b Research Initiative for Supra-Materials (RISM), Shinshu University 4-17-1 Wakasato Nagano 380-8553 Japan; c Research Center for Advanced Science and Technology, The University of Tokyo 4-6-1 Komaba, Meguro-ku Tokyo 153-8904 Japan; d Office of University Professors, The University of Tokyo 7-3-1 Hongo, Bunkyo-ku Tokyo 113-8656 Japan

## Abstract

Recombination of photoexcited carriers at interface states is generally believed to strongly govern the photoelectrochemical (PEC) performance of semiconductors in electrolytes. Sacrificial reagents (*e.g.*, methanol or Na_2_SO_3_) are often used to assess the ideal PEC performance of photoanodes in cases of minimised interfacial recombination kinetics as well as accelerated surface reaction kinetics. However, varying the sacrificial reagents in the electrolyte means simultaneously changing the equilibrium potential and the number of electrons required to perform the sacrificial reaction, and thus the thermodynamic and kinetic aspects of the PEC reactions cannot be distinguished. In the present study, we propose an alternative methodology to experimentally evaluate the energy levels of interfacial recombination centres that can reduce PEC performance. We prepare nonaqueous electrolytes containing three different Ru complexes with different bipyridyl ligands; redox reactions of Ru complexes represent one-electron processes with similar charge transfer rates and diffusion coefficients. Therefore, the Ru complexes can serve as a probe to isolate and evaluate only the thermodynamic aspects of PEC reactions. Recombination centres at the interface between a nonaqueous electrolyte and a Zn_0.25_Cd_0.75_Se particulate photoanode are elucidated using this method as a model case. The energy level at which photocorrosion proceeds is also determined.

## Abstract 2

Energy levels of interfacial recombination centres and photocorrosion potential for Zn_0.25_Cd_0.75_Se particulate photoanodes in nonaqueous electrolytes containing various Ru complexes are elucidated. Redox reaction of Ru complexes can serve as a probe.

## Graphical abstract

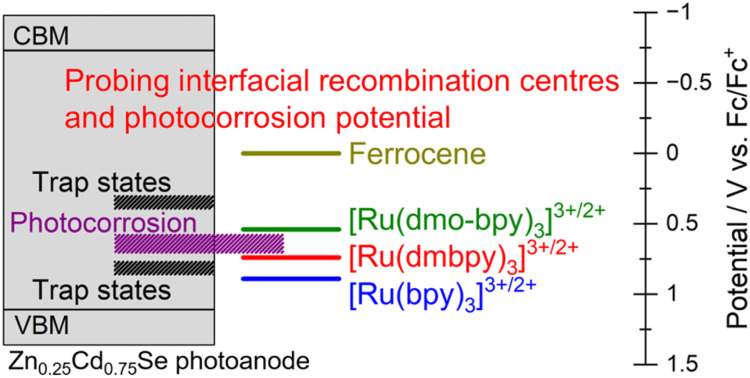

## Introduction

**Fig. 1:**
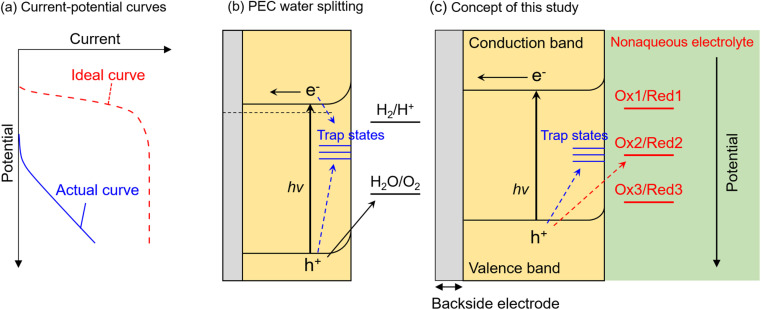
Schematic illustrations of (a) ideal and actual current–potential curves, (b) recombination of photogenerated carriers at interface trap states during PEC water splitting, and (c) concept of nonaqueous photoelectrochemistry using Ru complexes as a probe in this study.

Photocatalytic and photoelectrochemical (PEC) water splitting have been intensively studied as a promising means of achieving artificial photosynthesis.^1–3^ Photocatalyst and photoelectrode materials have typically been developed according to the guideline that a semiconductor for which the conduction band minimum (CBM) and valence band maximum (VBM) straddle the electrochemical potentials for hydrogen evolution and oxygen evolution should be capable of overall water splitting.^4,5^ However, there have been few reports of visible-light-responsive photocatalytic materials capable of performing overall water splitting *via* a one-step photoexcitation process (that is, by using a single photocatalyst component) in a powder suspension system,^6–10^ even if they possess a suitable band structure. Furthermore, if an n-type semiconductor with the required band structure for water splitting, that is the CBM locating more negative than the equilibrium potential for hydrogen evolution (0 V *vs.* reversible hydrogen electrode (RHE)) (or that which is actually capable of driving overall water splitting), is immersed in an aqueous electrolyte as a photoanode, the onset potential for the anodic photocurrent would also be expected to be more negative than 0 V_RHE_. However, most visible-light-responsive photoanodes exhibit a significantly more positive onset potential than 0 V_RHE_, as well as a low fill-factor in the current–potential curve (Fig. 1a), despite the CBM position being more negative than 0 V_RHE_.^11–14^ This inconsistency between ideal and actual photocatalytic performance is considered to be attributed to the recombination of photoexcited electrons and holes at interface states (Fig. 1b). That is, when interface states are located at a more positive or negative potential than that for the target reduction or oxidation reaction, the respective photoexcited electrons or holes are preferentially captured by the trap states and recombine with each other rather than with the target reactant in the electrolyte.^15–17^ Nevertheless, there is no versatile method to precisely elucidate the energy levels of recombination centres that limit PEC performance. In addition, photocorrosion of visible-light-responsive photocatalytic materials also seriously hinders stable progress of water splitting.^18^

A commonly used method for studying the recombination of photoexcited carriers at defects or interface states is time-resolved or transient absorption spectroscopy.^19–22^ However, although this is a powerful means of determining the dynamics of photoexcited carriers in a semiconductor, it does not necessarily elucidate the energy levels of recombination centres that actually govern photocatalytic and PEC performance. Additionally, such physical processes in semiconductors generally proceed on short timescales (from femtoseconds to microseconds), while actual catalytic processes also involve phenomena that occur on relatively long timescales (from microseconds to seconds)^23^ and thus are incapable of being detected by spectroscopic methods that focus on short-term effects. A PEC impedance spectroscopy method such as intensity-modulated photocurrent spectroscopy (IMPS) is capable of analysing charge-carrier dynamics on the different timescales involved in PEC reactions, especially for materials such as hematite (α-Fe_2_O_3_) that exhibit strong surface-charge recombination.^24^ However, this technique is incapable of elucidating the energy levels of trap states. Another possible approach to assess PEC performance is utilisation of sacrificial reagents such as methanol or Na_2_SO_3_.^25,26^ Because the thermodynamic onset potential for the decomposition of such reagents is much more negative than that for oxygen evolution (1.23 V_RHE_) and possibly than that for corrosion, a photoanode involved in the sacrificial reaction rather than the thermodynamically and kinetically slow oxygen evolution reaction is expected to generate an ideal photocurrent such that interfacial recombination and photocorrosion is minimised and the surface reaction kinetics are maximised (*i.e.*, the photocurrent is maximised).^27,28^ Indeed, the ratio of the photocurrent during oxygen evolution to that during a sacrificial reaction is sometimes referred to as the charge injection efficiency in the literature.^29–32^ However, different sacrificial reactions exhibit different equilibrium potentials and involve different numbers of electrons. For instance, oxygen evolution from water is a four-electron oxidation process, while oxidation of methanol or a sulphite anion to formaldehyde or a sulphate anion respectively proceeds *via* a two-electron oxidation process.^33,34^ In addition, these reactions require inner-sphere electron transfer, and thus the associated reaction mechanisms are complex. Therefore, a comparison between the PEC oxygen evolution reaction and other sacrificial reactions cannot distinguish between the thermodynamic and kinetic aspects of the PEC reaction.

In this study, we propose an alternative methodology to experimentally elucidate the energy levels of interfacial recombination centres limiting the PEC performance of a photoanode, as well as the energy level at which photocorrosion proceeds, by using nonaqueous electrolytes containing three different types of Ru complexes with different bipyridyl ligands (Fig. 1c).^35–38^ Reversible one-electron redox reactions of different Ru complexes proceed through outer-sphere electron transfer mechanisms with similar charge transfer rates and diffusion coefficients; the only difference between the Ru complexes is the thermodynamic equilibrium potential.^35,38^ Therefore, such complexes can serve as a probe to isolate and evaluate only the thermodynamic aspects of PEC reactions. That is, the photogenerated holes preferentially oxidise the reductant if the equilibrium redox potential is more negative than the potential for the trap states, whereas holes are trapped by interface states if the redox potential is more positive than the potential for the trap states. This methodology is expected to enable evaluation of the impact of the thermodynamic (not kinetic) parameters of the electrolyte on the behaviour of photogenerated holes inside the semiconductor as well as at the interface. Indeed, some redox reactions have been utilised as a probe for electrochemical surface analyses, such as scanning electrochemical microscopy.^39,40^ Instead, during the present nonaqueous PEC measurements, parameters such as the photocurrent flow as an indicator of the reaction rate, and the photoelectrode potential under quasi-equilibrium conditions during light irradiation (*i.e.*, open-circuit potential (OCP)), can be monitored as functions of the electrode potential and/or the light intensity. Additionally, since a nonaqueous electrolyte possesses a wider potential window than an aqueous electrolyte,^41^ the photocurrent originating from the decomposition of the solvent can be eliminated in the present nonaqueous system. A particulate Zn_0.25_Cd_0.75_Se photoanode is employed as a model visible-light-responsive photoanode. Although Zn_0.25_Cd_0.75_Se is incapable of generating oxygen from water due to photocorrosion, this material has been reported to be capable of generating an anodic photocurrent in a nonaqueous electrolyte containing a Ru 2,2′-bipyridine complex ([Ru(bpy)_3_]^3+/2+^).^42^ Additionally, this material can also be applied to various applications related to solar energy harvesting, such as hydrogen-evolving photocatalysts in a Z-scheme water splitting system^43^ or photovoltaics.^44^ Meanwhile, the results of our previous study also implied that the surface of the particulate Zn_*x*_Cd_1−*x*_Se photoanode might be defective.^42^ Thus, this material should be a suitable model for the first trial of the present analytical method to simultaneously study the energy levels of recombination centres and photocorrosion. The experimental details are described in the ESI (Fig. S1–S3†). The light-intensity and the electrode-potential dependence of the OCP and the incident-photon-to-current conversion efficiency (IPCE) for a Zn_0.25_Cd_0.75_Se photoanode in an acetonitrile electrolyte containing [Ru(bpy)_3_]^3+/2+^, Ru 4,4′-dimethyl-2,2′-bipyridine complex ([Ru(dmbpy)_3_]^3+/2+^),^37^ Ru 4,4′-dimethoxy-2,2′-bipyridine complex ([Ru(dmo-bpy)_3_]^3+/2+^),^36^ or ferrocene successfully elucidated the energy structure of the interface trap states. In addition, the energy level at which photocorrosion occurs was also determined using a combination of nonaqueous PEC measurements and detailed *ex situ* characterisation.

## Results and discussion

**Fig. 2:**
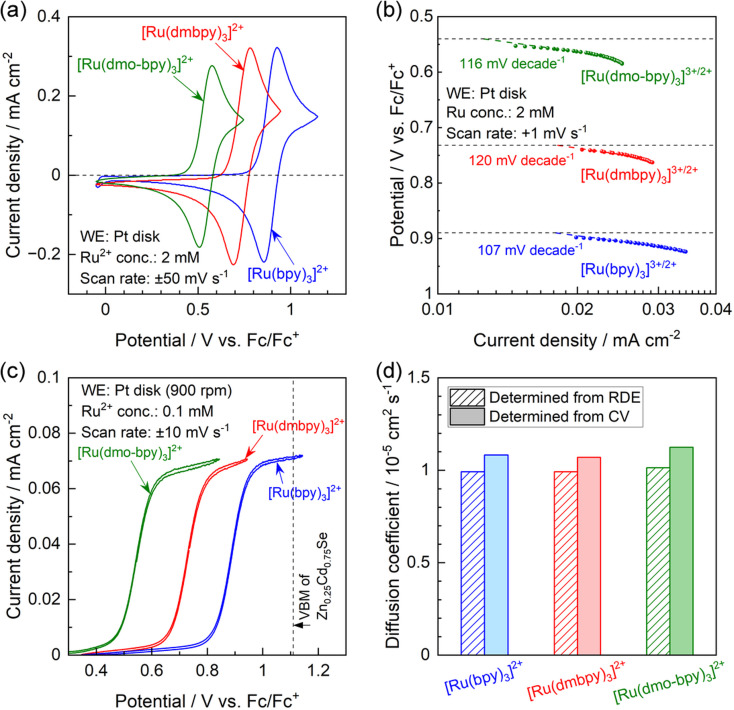
(a) CVs for a Pt disk electrode in an acetonitrile electrolyte containing 2 mM Ru^2+^ complexes and 0.1 M tetrabutylammonium hexafluorophosphate (TBAPF_6_). (b) Tafel plots obtained using nonaqueous electrolytes containing equimolar amounts of Ru^2+^ and Ru^3+^ complexes with a total concentration of 2 mM and 0.1 M supporting electrolyte. (c) CVs for a Pt rotating disk electrode (RDE) with a rotation speed of 900 rpm in a nonaqueous electrolyte containing 0.1 mM Ru^2+^ complexes and 0.1 M supporting electrolyte. (d) Summary of diffusion coefficients for Ru^2+^ complexes determined by Levich plots obtained from the results of RDE measurements and by the Randles–Sevcik equation obtained by varying the scan rate during CV measurements.

The results of physical characterisation (nuclear magnetic resonance (NMR) and high-resolution mass spectrometry) of the Ru complexes are provided in Fig. S4 in the ESI.† Cyclic voltammograms (CVs) obtained using a Pt disk electrode in acetonitrile electrolytes containing various Ru^2+^ complexes are presented in Fig. 2a. In all cases, the Pt electrode generated oxidation and reduction peaks with a peak separation of 68–92 mV, indicating that all Ru complexes showed reversible redox behavior.^45^ The redox equilibrium potential estimated from the midpoint between the oxidation and reduction peaks gradually became more negative as the electron-donating nature of the ligand increased; the redox potentials for [Ru(bpy)_3_]^3+/2+^, [Ru(dmbpy)_3_]^3+/2+^, and [Ru(dmo-bpy)_3_]^3+/2+^ were respectively 0.89, 0.74, and 0.54 V *vs.* the equilibrium potential for ferrocene and the ferrocenium ion (V *vs.* Fc/Fc^+^).^35,38^ Tafel plots were acquired using acetonitrile electrolytes containing equimolar amounts of Ru^2+^ and Ru^3+^, which were prepared by electrolysis of Ru^2+^ solutions at each equilibrium potential,^42,46^ as summarised in Fig. 2b. Assuming that the charge transfer coefficient is 0.5, the Tafel slope for the one-electron redox reaction is 118 mV per decade.^47^ All Ru complexes showed Tafel slopes close to this value, consistent with smooth one-electron redox behaviour. The redox reactions of [Ru(bpy)_3_]^3+/2+^, [Ru(dmbpy)_3_]^3+/2+^, and [Ru(dmo-bpy)_3_]^3+/2+^ on the Pt electrode showed similar exchange-current densities, indicating similar rate constants. Hydrodynamic voltammetry was performed using a Pt rotating disk electrode (RDE) in an electrolyte containing various Ru^2+^ complexes, and the results are presented in Fig. 2c. The oxidation currents converged towards the diffusion-limited current irrespective of the type of Ru complex, implying similar diffusion coefficients. When the VBM position for the semiconductor is relatively negative and therefore close to the equilibrium potential for the target reaction, the driving force for photogenerated holes may be insufficient to drive the reaction.^48,49^ In the present case, the oxidation current for [Ru(bpy)_3_]^2+^, which possesses the most positive equilibrium potential among the specimens studied, already reached the diffusion limit plateau at the VBM position for Zn_0.25_Cd_0.75_Se.^42^ This indicates that the Zn_0.25_Cd_0.75_Se photocatalysts exhibit a sufficient driving force to oxidise the present Ru complexes. The diffusion coefficients for the Ru complexes were further quantified using Levich plots^50^ (Fig. S5;† obtained by varying the RDE rotation speed) and by the Randles–Sevcik equation^45^ (Fig. S6;† obtained by varying the scan rate during CV measurements), with the results summarised in Fig. 2d. The two different methods gave similar values regardless of the type of Ru complex; all the present redox species exhibited a diffusion coefficient of approximately 1 × 10^−5^ cm^2^ s^−1^. The electrochemical parameters discussed above are summarised in Table 1. The electrochemical measurements indicate that the Ru complexes possess similar kinetic parameters (reaction rate constants and diffusion coefficients), and thus the only difference among them is the thermodynamic equilibrium potential.

**Table 1:** Electrochemical parameters for Ru complexes. Half-wave potential (*E*_1/2_)^a^, Tafel slope, exchange current density (*i*_0_), and diffusion coefficients (*D*_RDE_ and *D*_CV_)^b^^,^^c^

	*E*_1/2_ a/V *vs.* Fc/Fc^+^	Tafel slope/mV per decade	*i*_0_/mA cm^−2^	*D*_RDE_ b/cm^2^ s^−1^	*D*_CV_ c/cm^2^ s^−1^
[Ru(dmo-bpy)_3_]^3+/2+^	0.54	116	1.26 × 10^−2^	1.01 × 10^−5^	1.12 × 10^−5^
[Ru(dmbpy)_3_]^3+/2+^	0.74	120	1.81 × 10^−2^	9.92 × 10^−6^	1.07 × 10^−5^
[Ru(bpy)_3_]^3+/2+^	0.89	107	1.80 × 10^−2^	9.92 × 10^−6^	1.08 × 10^−5^

a Midpoint between the oxidation and reduction peaks in CV.

b Determined by RDE measurements.

c Determined by changing the scan rate during CV measurements.

**Fig. 3:**
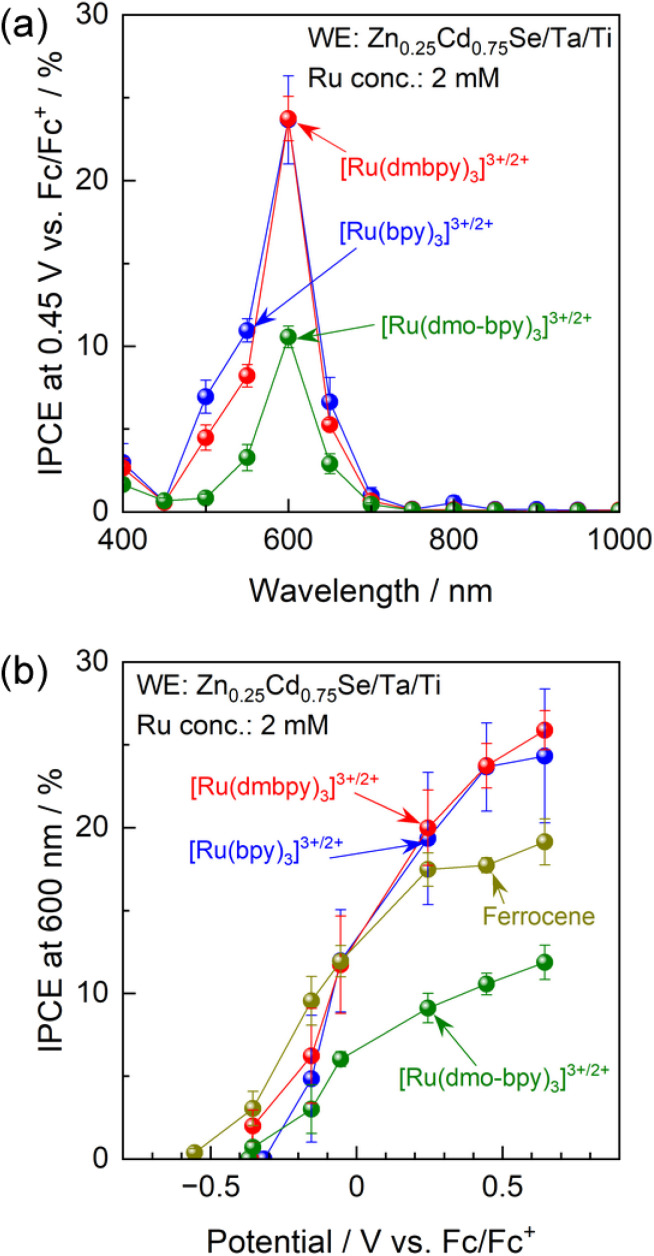
(a) IPCE spectra of the Zn_0.25_Cd_0.75_Se photoanode under an applied potential of 0.45 V *vs.* Fc/Fc^+^, and (b) IPCE–potential curves for the photoanode under illumination by 600 nm monochromatic light emitted from a Xe lamp. The acetonitrile electrolyte contained equimolar amounts of Ru^2+^ and Ru^3+^ complexes with a total concentration of 2 mM and 0.1 M TBAPF_6_.

IPCE spectra of the Zn_0.25_Cd_0.75_Se photoanode in acetonitrile electrolytes containing various Ru complexes with Ru^2+^ : Ru^3+^ molar ratios of unity are shown in Fig. 3a. Characterisation results obtained using scanning electron microscopy (SEM), diffuse reflectance spectroscopy (DRS), X-ray diffraction (XRD), and PEC measurements under simulated sunlight for Zn_0.25_Cd_0.75_Se particles are shown in Fig. S7–S9.† The onset wavelength for anodic photocurrent production was around 700 nm and agreed well with the absorption edge for the Zn_0.25_Cd_0.75_Se particles. The Ru complexes showed strong light absorption at 400–500 nm (Fig. S10†),^51–54^ which hinders light absorption by the semiconductor and thus causes a drastic decrease in IPCE in this wavelength region. The above results suggest that photoexcitation of the Ru complexes did not contribute to the PEC reaction and that the observed photocurrent is solely attributed to one-step bandgap photoexcitation of Zn_0.25_Cd_0.75_Se. Photoanodes combined with [Ru(bpy)_3_]^3+/2+^ or [Ru(dmbpy)_3_]^3+/2+^ showed similar IPCE spectra (IPCE of approximately 23.7% under 600 nm monochromatic light), while the IPCE value decreased to 10.6% when a [Ru(dmo-bpy)_3_]^3+/2+^ redox was employed. The dependence of the IPCE on the electrode potential demonstrates that the onset potential for the photoanode in the nonaqueous electrolyte containing Ru complexes is approximately −0.4 V *vs.* Fc/Fc^+^, irrespective of the equilibrium potential for the redox (Fig. 3b). Meanwhile, the photocurrents generated by the photoanode combined with [Ru(bpy)_3_]^3+/2+^ or [Ru(dmbpy)_3_]^3+/2+^ were much larger than the case of the [Ru(dmo-bpy)_3_]^3+/2+^ redox over the entire potential range. For comparison, the PEC performance of the photoanode in a nonaqueous electrolyte containing ferrocene, which possesses a much more negative equilibrium potential than the Ru complexes, was also measured. The photocurrent originating from the oxidation of ferrocene appeared at a more negative potential than that for [Ru(bpy)_3_]^3+/2+^ or [Ru(dmbpy)_3_]^3+/2+^, but was slightly lower at positive potentials. Here, it should be noted that the concentration of ferrocene had little effect on the anodic photocurrent (Fig. S11†), and thus the total concentration of redox species (Ru complexes and ferrocene) was fixed at 2 mM in subsequent experiments. It was also confirmed that the observed photocurrent was not limited by diffusion processes (Fig. S12†). The facts that the one-electron redox process for Ru complexes is smooth and reversible and that the kinetic parameters involved in the redox reactions are almost identical indicate that the photocurrent should reflect charge separation in the semiconductor. The following generalisations should be noted: (1) if the photocurrent is determined solely by charge separation in the semiconductor, the thermodynamic equilibrium potential for the reactant in the electrolyte should no longer affect the photocurrent; (2) if the n-type semiconductor contains recombination centres that have a more negative potential than the equilibrium potential for the reactant in the electrolyte, a certain percentage of the photoexcited carriers would be trapped and the remaining holes would take part in the PEC reaction, resulting in a decrease in photocurrent;^15–17^ and (3) therefore, if the photocurrent is sensitive to the redox species employed, a higher photocurrent should be obtained when using a redox shuttle possessing a more negative thermodynamic equilibrium potential. However, the present observations that the highest photocurrent was obtained when using [Ru(bpy)_3_]^3+/2+^, which has the most positive equilibrium potential, appear to contradict this.

**Fig. 4:**
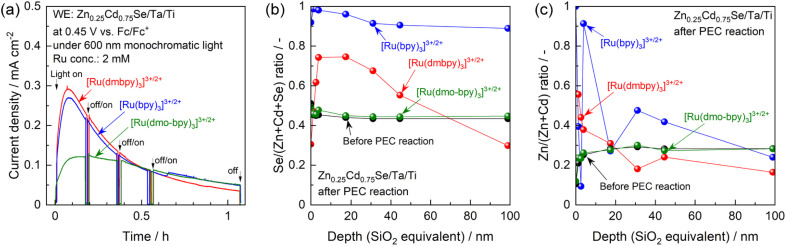
(a) Current–time curves for the Zn_0.25_Cd_0.75_Se photoanode in the acetonitrile electrolyte containing equal molar amounts of Ru^2+^ and Ru^3+^ complexes with the total concentration of 2 mM and 0.1 M TBAPF_6_ under illumination of 600 nm monochromatic light emitted from a Xe lamp. (b) Se/(Zn + Cd + Se) and (c) Zn/(Zn + Cd) molar ratios for the photoanode before and after the long-term PEC reaction determined by XPS.

One possible reason for this apparent contradiction is the competing effect of photocorrosion, that is, photogenerated holes might be consumed by both oxidation of Ru complexes and self-oxidation. Indeed, the photocurrent generated by the photoanode in the electrolyte containing [Ru(bpy)_3_]^3+/2+^ or [Ru(dmbpy)_3_]^3+/2+^ drastically decreased under prolonged light irradiation, compared with the case of [Ru(dmo-bpy)_3_]^3+/2+^ (Fig. 4a). After prolonged illumination, the photocurrent values finally became almost completely insensitive to the redox potentials of Ru complexes. This implies that PEC oxidation of [Ru(bpy)_3_]^3+/2+^ or [Ru(dmbpy)_3_]^3+/2+^ competed with photocorrosion. Compositional depth profiles for the photoanodes before and after the PEC reactions were obtained using X-ray photoelectron spectroscopy (XPS), and the results are presented in Fig. 4b and c. The near-surface region of the as-prepared specimen (before the PEC reaction) to a depth of a few nanometres contained slightly larger amounts of Se and smaller amounts of Zn compared to the stoichiometric values, while the bulk of the photocatalyst particles retained a stoichiometric composition. However, after the PEC reaction using [Ru(bpy)_3_]^3+/2+^ or [Ru(dmbpy)_3_]^3+/2+^ as a redox shuttle, the Se/(Zn + Cd + Se) and Zn/(Zn + Cd) ratios to a depth of 100 nm from the surface drastically increased. For [Ru(bpy)_3_]^3+/2+^ with the most positive equilibrium potential, the variation in composition was more prominent than that for [Ru(dmbpy)_3_]^3+/2+^. Cross-sectional SEM observations demonstrated that, after the PEC reaction using [Ru(bpy)_3_]^3+/2+^, the near-surface region of the photocatalyst particles was damaged to a depth on the order of microns (Fig. S13†). It is generally considered that the photogenerated holes oxidise Se^2−^ in the crystal lattice during the photocorrosion process, resulting in the precipitation of metal-like Se at the surface and elution of metal cations into the electrolyte.^55^ Therefore, the near-surface variations in the composition of Zn_0.25_Cd_0.75_Se after the PEC reaction observed in the present study lead to the conclusions that Se was precipitated at the surface as a result of photocorrosion, that the Cd species might be more photocorrosive than the Zn species, and that [Ru(bpy)_3_]^3+/2+^ caused more serious photocorrosion than [Ru(dmbpy)_3_]^3+/2+^. Here, analysis of the electrolyte after the PEC reaction suggested that photocorrosion occurred only at the surface and was not significant enough to change the composition of the electrolyte (details given in Fig. S14†). Indeed, it was roughly estimated that the faradaic efficiency for photocorrosion competing with the oxidation of [Ru(dmbpy)_3_]^2+^ should be less than 20%, and that the amount of eluted Zn_0.25_Cd_0.75_Se was equivalent to only 0.3–0.5% of the total number of moles of Ru complex contained in the electrolyte (details given in Fig. S15 and Table S1†). Interestingly, the composition of the photoanode surface after the PEC reaction in the electrolyte containing [Ru(dmo-bpy)_3_]^3+/2+^ was almost entirely unchanged, implying that little photocorrosion occurred by PEC oxidation of [Ru(dmo-bpy)_3_]^2+^; this is also confirmed by cross-sectional SEM observations (Fig. S13†). The XPS peaks assigned to Zn 2p and Cd 3d were slightly shifted to higher binding energies after the PEC reaction employing [Ru(bpy)_3_]^3+/2+^ or [Ru(dmbpy)_3_]^3+/2+^, while they were identical to those for the as-prepared specimen in the case of [Ru(dmo-bpy)_3_]^3+/2+^ (Fig. S16†). The Se 3d XPS spectra also showed that the surface of the photoanode in an electrolyte containing [Ru(bpy)_3_]^3+/2+^ or [Ru(dmbpy)_3_]^3+/2+^ was covered with a metal-like Se species, while there was no change in the chemical state of Se for the case of [Ru(dmo-bpy)_3_]^3+/2+^ (Fig. S16†). These observations also support the resistance of the photoanode to photocorrosion when employing [Ru(dmo-bpy)_3_]^3+/2+^. After a long-term PEC reaction, the IPCE for a photoanode in an electrolyte containing [Ru(bpy)_3_]^3+/2+^ or [Ru(dmbpy)_3_]^3+/2+^ was dramatically reduced over the entire potential range, but was almost entirely unchanged for the case of [Ru(dmo-bpy)_3_]^3+/2+^ or ferrocene (Fig. S17†). Consequently, the photoanode generated an almost identical photocurrent irrespective of the redox equilibrium potential for the Ru complex. This means that the photocurrent obtained using the electrolyte containing [Ru(bpy)_3_]^3+/2+^ or [Ru(dmbpy)_3_]^3+/2+^, as shown in Fig. 3, is partially attributed to photocorrosion, and thus the photocurrent originating solely from oxidation of Ru^2+^ is not affected by the thermodynamic equilibrium potentials for the Ru complexes. It can also be concluded that a more positive equilibrium potential results in more significant photocorrosion, and that the threshold potential for photocorrosion is between the equilibrium potentials for [Ru(dmo-bpy)_3_]^3+/2+^ and [Ru(dmbpy)_3_]^3+/2+^ (approximately 0.54–0.74 V *vs.* Fc/Fc^+^). It has been reported that the equilibrium potential for anodic corrosion of metal chalcogenides such as ZnS, CdS, ZnSe, and CdSe in aqueous media is generally around 0.5 V relative to the standard hydrogen electrode.^56–59^ This value is roughly equivalent to 0 V *vs.* Fc/Fc^+^, and is thus more negative than the value measured in the acetonitrile electrolyte in the present study. The Gibbs free energies for Cd^2+^ and Zn^2+^ transfer from water to acetonitrile have been reported to be 12–42 and 26–67 kJ mol^−1^, respectively,^60,61^ indicating that these cations are more stable in water than in acetonitrile. The instability of the cations in the nonaqueous electrolyte might result in a positive shift of the corrosion potential thermodynamically and/or kinetically. Here, it should be noted that the photoanode exhibited a larger photocurrent and a more negative onset potential for ferrocene than for the Ru complexes (Fig. S17†), implying the existence of interface trap states with a potential intermediate between the equilibrium potentials for ferrocene and [Ru(dmo-bpy)_3_]^3+/2+^.

**Fig. 5:**
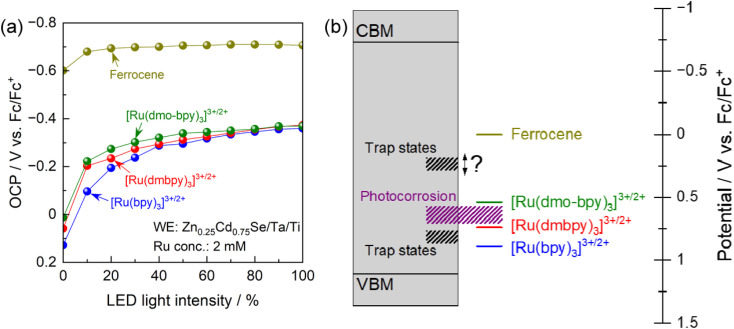
(a) OCPs of the Zn_0.25_Cd_0.75_Se photoanode in the acetonitrile electrolyte containing equal molar amounts of Ru^2+^ and Ru^3+^ complexes with the total concentration of 2 mM and 0.1 M TBAPF_6_ as functions of light intensity. Various intensities of 600 nm monochromatic light from the LED were used as light sources. (b) Schematic energy diagram of the band structure of Zn_0.25_Cd_0.75_Se accompanied by the potentials for trap sites and photocorrosion.

To elucidate the trap states in more detail, the OCP for the photoanode in electrolytes containing various redox shuttles is plotted as a function of the light intensity in Fig. 5a. As shown in Fig. S3,† the light intensity obtained from the LED used for these measurements was smaller than that for the Xe lamp used for the PEC measurements (Fig. 3 and 4). It is generally considered that in the presence of interface sites acting as recombination centres, the OCP for the photoanode becomes more negative as the light intensity increases because the trap states are filled with an increasing number of photogenerated holes.^62,63^ More negative OCP means a more ideal situation in which the electrode potential of the photoanode is close to the flat-band condition, indicating less recombination losses. Under weak illumination, the OCP for the photoanode in electrolytes containing Ru complexes showed drastic light-intensity dependence at a relatively positive potential region (−0.2 to 0.1 V *vs.* Fc/Fc^+^). For [Ru(bpy)_3_]^3+/2+^, the dependence of the OCP on the light intensity was particularly strong under weak light illumination up to 40% relative light intensity. Meanwhile, under intense illumination, the OCPs converged to a constant value of approximately −0.37 V *vs.* Fc/Fc^+^ irrespective of the equilibrium potential for the Ru complex. These results suggest the presence of recombination centres with a potential intermediate between the equilibrium potentials for [Ru(dmbpy)_3_]^3+/2+^ and [Ru(bpy)_3_]^3+/2+^, in addition to those at a more negative potential than the equilibrium potential for [Ru(dmo-bpy)_3_]^3+/2+^. The latter trap sites would be expected to cause a larger reduction in PEC performance. An interesting point is that the OCP for the photoanode in ferrocene was quite close to the CBM for Zn_0.25_Cd_0.75_Se,^42^ and showed only a minimal light-intensity dependence. This indicates that there were no trap states at potentials more negative than the equilibrium potential for ferrocene. Fig. 5b provides a summary of the results obtained here for trap sites and photocorrosion. It would be of interest to determine a more precise value for the potential for trap states between the equilibrium potentials for ferrocene and [Ru(dmo-bpy)_3_]^3+/2+^.

**Fig. 6:**
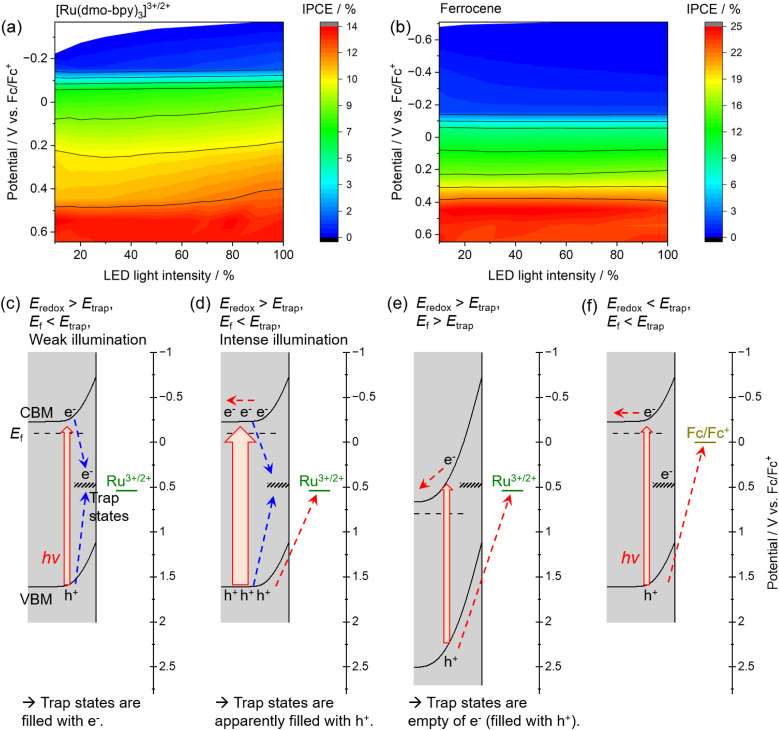
IPCE as a function of electrode potential and light intensity for the Zn_0.25_Cd_0.75_Se photoanode in an acetonitrile electrolyte containing (a) equimolar amounts of Ru^2+^ and Ru^3+^ complexes with a total concentration of 2 mM or (b) 2 mM ferrocene. 0.1 M TBAPF_6_ was used as a supporting electrolyte. The intensity of the 600 nm LED light source was varied in the experiments. (c–f) Mechanisms explaining the electrode-potential and light-intensity dependence of IPCE.

The dependence of the IPCE on the electrode potential and the light intensity in the case of [Ru(dmo-bpy)_3_]^3+/2+^ and ferrocene is shown in Fig. 6. When [Ru(dmo-bpy)_3_]^3+/2+^ was employed (Fig. 6a), the IPCE exhibited a remarkable light-intensity dependence at relatively negative potentials, that is, larger IPCE values were obtained under more intense light irradiation. When the electrode potential was more positive than approximately 0.5 V *vs.* Fc/Fc^+^, the IPCE was almost completely independent of the light intensity. This means that the critical trap sites determining the PEC performance are located at this potential. Using ferrocene, the IPCE did not exhibit a significant light-intensity dependence over almost the entire potential range, and was larger than that using [Ru(dmo-bpy)_3_]^3+/2+^ (Fig. 6b). This is consistent with the absence of trap sites at potentials more negative than the ferrocene redox potential, as discussed in the previous paragraph. Typically, when the trap sites locate at the energy level between the Fermi level and CBM of the n-type semiconductor (that is, the trap sites are devoid of electrons), increasing the light intensity might result in reduced IPCEs due to accelerated trap-assisted recombination,^64^ or possibly result in enhanced photoconductivity.^65^ In contrast, when the trap sites exist near the centre of the bandgap and thus at an energy level more positive than the Fermi level of the n-type semiconductor (that is, the trap sites are intrinsically filled with electrons), the IPCEs can be enhanced as the light intensity increases.^64^ In our present case, the increasing of IPCEs of the Zn_0.25_Cd_0.75_Se photoanode as the light intensity increases should be consistent with the existence of deep trap sites. The mechanism causing the dependence of the IPCE on the electrode potential and the light intensity in the case of [Ru(dmo-bpy)_3_]^3+/2+^ or ferrocene can be explained as follows. Here, it should be noted that, if charge carriers enormously accumulate at the photoanode surface due to extremely positive applied potential and/or quite slow surface reaction kinetics under light illumination, the band edges might shift to positive potentials.^66^ In the present case, because smooth one-electron redox is employed and thus charge carriers might not accumulate at the surface, we assume that the band edge potentials are fixed. When the electrode potential (that is, the Fermi level for the semiconductor, *E*_f_) is more negative than the potential for the interface trap sites (*E*_trap_), the trap sites are filled with electrons. In such a case, when the photoanode is irradiated by low-intensity light in the electrolyte containing [Ru(dmo-bpy)_3_]^3+/2+^, the majority of the photogenerated holes are captured by trap sites with a more negative potential than the equilibrium potential for the Ru complex (*E*_redox_) (Fig. 6c). As the light intensity increases, the trap states are always filled with holes, so that the interface sites are seemingly devoid of electrons and are deactivated,^62,63^ resulting in an increase in the percentage of photogenerated carriers that can contribute to oxidation of the Ru complex (Fig. 6d). This is considered to be the origin of the light-intensity dependence of the IPCE under a relatively negative electrode potential when using [Ru(dmo-bpy)_3_]^3+/2+^. When a potential more positive than that for the trap states is applied to the photoanode, the majority carriers (electrons) are not resident at interface sites, and thus the sites no longer serve as trapping centres for the photogenerated holes (Fig. 6e). Consequently, the photoanode combined with [Ru(dmo-bpy)_3_]^3+/2+^ does not show a light-intensity dependence at potentials more positive than *E*_trap_ (0.5 V *vs.* Fc/Fc^+^). Meanwhile, because the equilibrium potential for ferrocene is significantly more negative than *E*_trap_, photogenerated holes become preferentially involved in the oxidation of ferrocene rather than being captured by the trap sites, irrespective of the applied potential or the incident light intensity (Fig. 6f). Therefore, the photoanode combined with ferrocene exhibits no light-intensity dependence over the entire potential range. Thus, the potential for recombination centres and the threshold potential for photocorrosion have been successfully determined. Interface trap sites exist around potentials of 0.74–0.89 V *vs.* Fc/Fc^+^ and 0.5 V *vs.* Fc/Fc^+^, and the latter have a particularly large effect on the PEC performance of the present Zn_0.25_Cd_0.75_Se photoanodes. In the case of II–VI chalcogenides, it has been reported that cation vacancy levels can lie in the lower half of the bandgap or near the centre of the bandgap.^67–71^ Therefore, the recombination centres observed in the present study might also be attributed to Zn^2+^ and/or Cd^2+^ vacancies. Typical PEC measurements using a sacrificial hole scavenger under either aqueous or nonaqueous conditions were incapable of precisely assessing the energy levels of some recombination centres and/or photocorrosion potential existing between the bandgap (Fig. S18 and S19†). Meanwhile, the techniques typically employed to detect such deep levels (near the centre of the bandgap) in semiconductor devices, such as deep-level transient spectroscopy or isothermal-capacitance transient spectroscopy,^72–74^ are not necessarily suitable for analysing particulate photocatalytic materials. Additionally, such methods cannot be used for materials that are unstable in the presence of stress induced by heat or bias voltages. We expect that the proposed concept based on nonaqueous photoelectrochemistry using a redox shuttle as a probe can be a novel tool for visualising the energy structures at the interface between photocatalyst particles and an electrolyte.

## Conclusions

The PEC performance of Zn_0.25_Cd_0.75_Se photoanodes in nonaqueous electrolytes containing different redox shuttles was precisely analysed. Various Ru bipyridyl complexes with identical kinetic parameters (reaction rate constants and diffusion coefficients) but different thermodynamic equilibrium potentials were prepared. The photoanode was capable of driving PEC oxidation of [Ru(dmo-bpy)_3_]^2+^ with a faradaic efficiency of almost unity, while PEC oxidation of [Ru(bpy)_3_]^2+^ or [Ru(dmbpy)_3_]^2+^ competed with photocorrosion with a threshold potential of around 0.54–0.74 V *vs.* Fc/Fc^+^. The dependence of the OCP and the IPCE on the light intensity and the electrode potential revealed that interface trap states acting as recombination centres existed at potentials between the equilibrium potentials for [Ru(dmbpy)_3_]^3+/2+^ and [Ru(bpy)_3_]^3+/2+^ (0.74–0.89 V *vs.* Fc/Fc^+^) and between the equilibrium potentials for ferrocene and [Ru(dmo-bpy)_3_]^3+/2+^ (0.5 V *vs.* Fc/Fc^+^). The latter trap sites were found to have a greater effect on the PEC performance.

The proposed method based on nonaqueous photoelectrochemistry is expected to offer an alternative approach to spectroscopic techniques or PEC measurements using sacrificial reagents. It is capable of determining the factors that govern PEC performance by separately evaluating only the thermodynamic aspects of PEC reactions. One of the most important reactions in the field of solar energy harvesting is hydrogen production using water as an electron source. Thus, future elucidation of the electronic structure of defect states contained in oxygen-evolving photoanodes should contribute to the designing of more efficient materials. Additionally, application of the present nonaqueous PEC measurements to the assessment of more complicated reaction systems involving several competing PEC processes, such as charge recombination, photocorrosion, decomposition of solvent (water), and redox reactions, will be of interest from the scientific aspect. Therefore, application of this method to photocatalytic materials that are actually capable of generating oxygen from water is the next challenge.

## Data availability

The data supporting the findings of this study are available within the paper and its ESI† files. All relevant data are available from the corresponding authors on request.

## Author contributions

Y. Kageshima conceptualised the research, established the methodology and wrote a draft of the manuscript. H. Takano synthesised the materials and performed the photoelectrochemical measurements. M. Nishizawa contributed to the photoelectrochemical measurements. F. Takagi contributed to the synthesis of Ru complexes. H. Kumagai contributed to the synthesis of Ru complexes and to the photoelectrochemical measurements. K. Teshima and K. Domen supervised the research. H. Nishikiori conceptualised and supervised the research and contributed to reviewing and editing the manuscript. All authors contributed to the research, discussed the results and approved the final version of the manuscript.

## Conflicts of interest

There are no conflicts to declare.

## Supplementary Material

SC-015-D4SC00511B-s001
