# Supplementary material for: Precise analyses of photoelectrochemical reactions on particulate Zn0.25Cd0.75Se photoanodes in nonaqueous electrolytes using Ru bipyridyl complexes as a probe
Source: Chem Sci. 2024 Apr 16;15(18):6679–89. doi: 10.1039/d4sc00511b (PMC11077565; doi:10.1039/d4sc00511b)
Supplement: SC-015-D4SC00511B-s001 [file SC-015-D4SC00511B-s001.pdf]

Electronic Supplementary Material (ESI) for Chemical Science.
This journal is © The Royal Society of Chemistry 2024

Precise analyses of photoelectrochemical reaction on particulate $Zn_{0.25}Cd_{0.75}Se$ photoanode in nonaqueous electrolyte using Ru bipyridyl complexes as probe

Yosuke Kageshima,*[ab] Hiroto Takano,[a] Mika Nishizawa,[a] Fumiaki Takagi,[a] Hiromu Kumagai,[c] Katsuya Teshima,[ab] Kazunari Domen,[bd] Hiromasa Nishikiori*[ab]

[a] Department of Materials Chemistry, Faculty of Engineering, Shinshu University, 4-17-1 Wakasato, Nagano 380-8553, Japan.

[b] Research Initiative for Supra-Materials (RISM), Shinshu University, 4-17-1 Wakasato, Nagano 380-8553, Japan.

[c] Research Center for Advanced Science and Technology, The University of Tokyo, 4-6-1 Komaba, Meguro-ku, Tokyo 153-8904, Japan.

[d] Office of University Professors, The University of Tokyo, 7-3-1 Hongo, Bunkyo-ku, Tokyo 113-8656, Japan.

Table of Contents

Experimental Procedures S3

Preparation of Ru complexes S3

Preparation of particulate $Zn_{0.25}Cd_{0.75}Se$ photoanodes S3

Characterisation S3

(Photo)electrochemical measurements S3

Results and Discussion S7

Characterisation of Ru complexes S7

Diffusion coefficients for Ru complexes S8

Characterisation of $Zn_{0.25}Cd_{0.75}Se$ photocatalyst particles S10

Absorption spectra of acetonitrile electrolyte containing Ru complexes S12

Influence of concentration of ferrocene on PEC performance S13

Comparison of photocurrent generated by $Zn_{0.25}Cd_{0.75}Se$ photoanodes and diffusion-limited current for redox shuttle S14

Change in morphology and composition of $Zn_{0.25}Cd_{0.75}Se$ photoanodes induced by photocorrosion S15

Ratio of Ru^{2+} to Ru^{3+} in bulk electrolyte during PEC reaction S15

Maximum expected degree of photocorrosion S16

XPS analysis of $Zn_{0.25}Cd_{0.75}Se$ photoanodes S18

Change in IPCE-potential curves after PEC reaction S19

PEC measurements using sacrificial reagents S20

References S21

Experimental Procedures

Preparation of Ru complexes

Tris(2,2'-bipyridine)ruthenium(II) hexafluorophosphate ($[Ru(bpy)_3](PF_6)_2$) was used as purchased.[1, 2] Tris(4,4'-dimethyl-2,2'-bipyridine)ruthenium(II) hexafluorophosphate ($[Ru(dmbpy)_3](PF_6)_2$) and tris(4,4'-dimethoxy-2,2'-bipyridine)ruthenium(II) hexafluorophosphate ($[Ru(dmo\text{-}bpy)_3](PF_6)_2$) were synthesised according to the procedure reported in the literature.[3, 4] $RuCl_3$ and 4,4'-dimethyl-2,2'-bipyridine were dissolved in ethanol with a $RuCl_3$: ligand molar ratio of 1 : 6.27 (typically, 0.13 mmol of the Ru salt and 0.815 mmol of the bipyridine ligand were dissolved in 20 mL of solvent), and were refluxed using an aluminum bead bath at 120 °C under N_2 atmosphere for 15 h. After the reflux, the solvent was removed by a rotary evaporator, and the resultant precipitates were dissolved in water. An aqueous solution containing 0.5 mmol NH_4PF_6 ($RuCl_3$: NH_4PF_6 molar ratio of 1 : 3.85) was added to the solution containing the Ru complex, and the aqueous mixture was stored in a refrigerator overnight. The recrystallised particles were collected by vacuum filtration. For the case of preparation of $[Ru(dmo\text{-}bpy)_3](PF_6)_2$, $RuCl_3$ and the ligand were refluxed in a 20-mL mixture of ethanol and water (ethanol : water volume ratio = 1 : 1) for 15 h.

Preparation of particulate $Zn_{0.25}Cd_{0.75}Se$ photoanodes

$Zn_{0.25}Cd_{0.75}Se$ particles were synthesised through a solid-state reaction in a sealed quartz ampule.[1] The ZnSe, CdSe, and Se powders were mixed in an Ar-filled glove box with Zn/(Zn+Cd) and Se/(Zn+Cd) molar ratios of 0.25 and 1.1, respectively. The mixture was sealed in a quartz ampule and heated at 600 °C for 15 h.

Photoanodes consisting of $Zn_{0.25}Cd_{0.75}Se$ particles were fabricated by the particle transfer (PT) method (Figure S1).[1, 5] The synthesised photocatalysts were coated on a primary glass substrate by drop-casting a suspension of particles in isopropanol and drying in air. A thin Ta contact layer and a thick Ti conductor layer were sequentially deposited on the photocatalyst layer by radiofrequency magnetron sputtering. During sputter-deposition of the backside metal electrode layer, the temperature of the glass substrate coated with the photocatalyst particles was kept at 200 °C. The assembly consisting of photocatalyst particles anchored on the metal layer was peeled from the primary glass substrate, and the excessive particles without direct contact to the metal layer were removed by sonication in water. The composite served as a photoanode after being fixed on a secondary glass substrate with epoxy resin.[6] A glass spacer with a thickness of approximately 1 mm was also attached to the photoanode to maintain a constant thickness of the colored electrolyte.

Characterisation

^1H nuclear magnetic resonance (NMR) spectra of the Ru complexes were recorded on a BRUKER AVANCE III Fourier 300 (300 MHz) spectrometer. Chemical shifts were expressed in parts per million downfield from tetramethylsilane (TMS) as an internal standard. High-resolution mass spectrometry (HRMS) of the Ru complexes was performed on a BRUKER micrOTOF II electrospray ionisation (ESI) – time-of-flight (TOF) spectrometer.

The morphologies of the $Zn_{0.25}Cd_{0.75}Se$ particulate photoanodes were examined using field-emission scanning electron microscopy (FE-SEM; Hitachi, SU8000) equipped with an energy-dispersive X-ray spectroscopy (EDS) apparatus. A diffuse reflectance (DR) spectrum of the photocatalyst particles and transmission spectra of the acetonitrile electrolyte containing Ru complexes were obtained using ultraviolet−visible-near-infrared spectroscopy (Jasco, V-770). The crystal structure of the $Zn_{0.25}Cd_{0.75}Se$ particles was characterised by X-ray diffraction (XRD; Rigaku, Miniflex600) using the Cu Kα line. Surface elemental compositions and depth profiles of the particulate photoanodes before and after photoelectrochemical (PEC) measurements were determined by X-ray photoelectron spectroscopy (XPS; Ulvac-Phi, PHI Quantera II) employing the Al Kα line.

(Photo)electrochemical measurements

(Photo)electrochemical measurements were conducted in a typical three-electrode setup using a commercially available Ag/Ag^+ electrode equipped with an acetonitrile electrolyte junction and a Pt black-coated Pt coil as reference and counter electrodes, respectively. Tetrabutylammonium hexafluorophosphate ($TBAPF_6$) was used as purchased as a supporting electrolyte. An acetonitrile electrolyte containing 0.1 M $TBAPF_6$ and equimolar amounts of Ru^{2+} and Ru^{3+} complexes with a total Ru concentration of 2 mM was prepared by electrochemical oxidation of Ru^{2+} complexes as shown in Figure S2a.[1, 2] During this process, an electrode potential equal to the equilibrium potential for the Ru complex was applied to a Pt mesh

electrode immersed in an acetonitrile electrolyte containing 0.1 M $TBAPF_6$ and 2 mM Ru^{2+} complex until the anodic current reached almost zero. Cyclic voltammograms (CVs) for the Ru complexes were acquired by using a commercial Pt disk electrode and rotating disk electrode (RDE) as the working electrode. Especially during the acquisition of the Tafel plots, the equilibrium potential for the bulk electrolyte was kept constant by continuous bulk electrolysis using the Pt mesh electrode (Figure S2b). The cell configuration during the PEC measurements is shown in Figure S2c. The photoanodes were irradiated through a layer of acetonitrile electrolyte with a thickness of approximately 1 mm. The electrolyte was purged using Ar and vigorously stirred during the PEC measurements. A 300-W Xe lamp equipped with a monochromator, a variable-output light-emitting diode (LED; Asahi spectra, CL), and simulated sunlight adjusted to AM 1.5G were used as light sources. The results of PEC measurements presented in Figures 3 and 4 in the main manuscript were obtained using the 300-W Xe lamp equipped with a monochromator, while the light-intensity dependence (Figures 5 and 6) was examined using the LED light source. The spectra of the 600-nm monochromatic light produced by the Xe lamp and LED are presented in Figure S3. Incident-photon-to-current conversion efficiencies (IPCEs) in this study (e.g., Figures 3 and 6 in the main manuscript) were calculated from photocurrent values measured by the chronoamperometry mode, rather than the continuous potential scan. Here, photocurrent approximately 3—5 s after the monochromatic light illumination started with applying a constant potential was employed, in order to avoid the contribution of non-faradaic current.

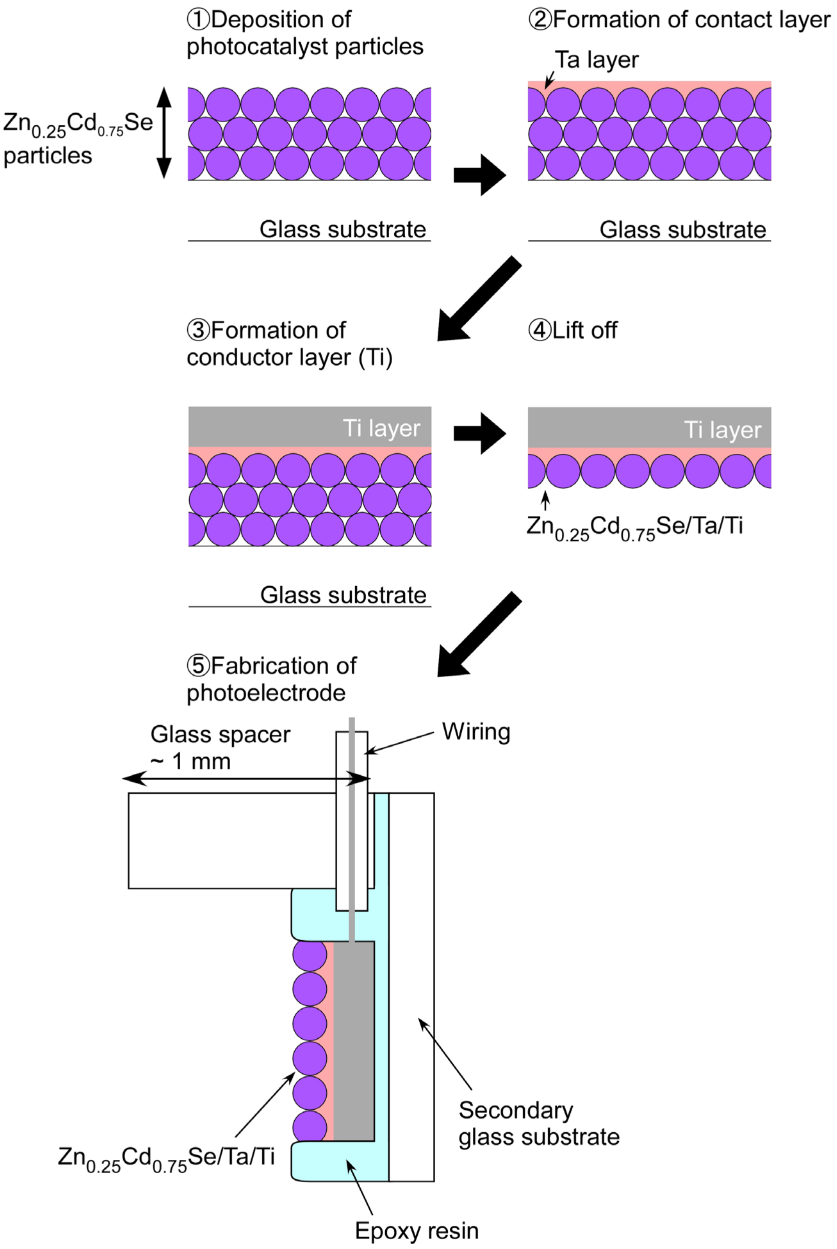

Figure S1. Schematic illustration of PT method to fabricate particulate $Zn_{0.25}Cd_{0.75}Se$ photoanodes.

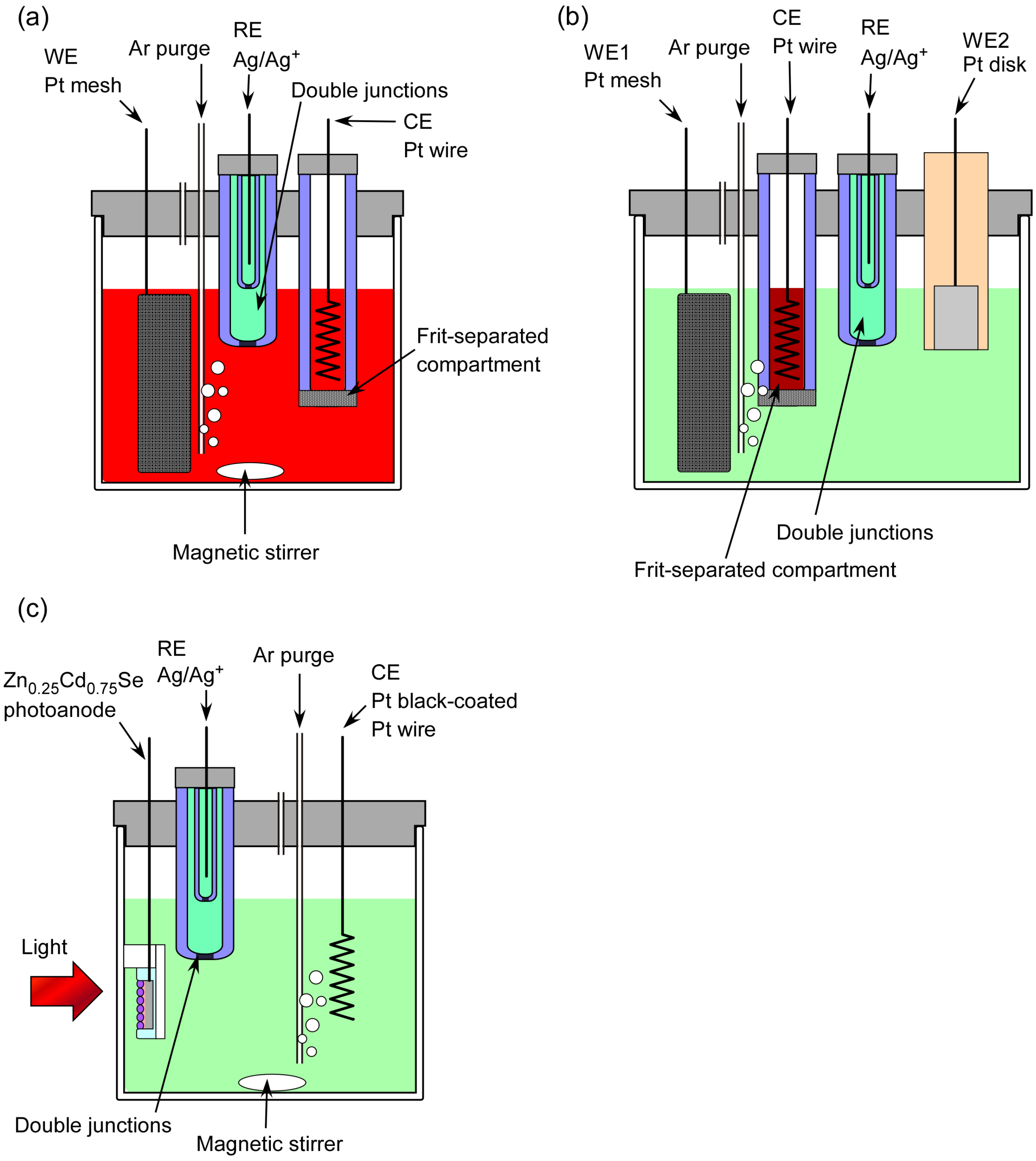

Figure S2. Schematic drawings of experimental setup for (a) bulk electrolysis oxidation of Ru^{2+} complex to Ru^{3+}, (b) electrochemical measurements for acquiring Tafel plots, and (c) PEC measurements.

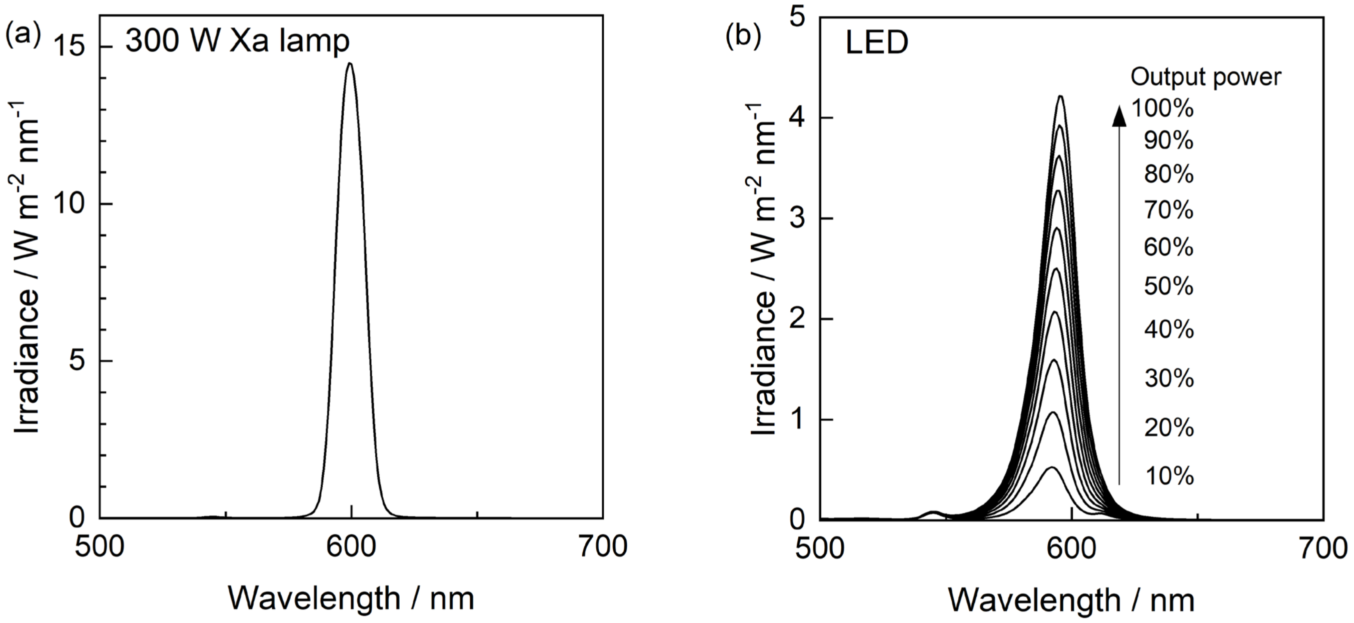

Figure S3. Spectra of 600-nm monochromatic light produced by (a) 300-W Xe lamp equipped with monochromator and (b) LED with various output intensities.

Results and Discussion

Characterisation of Ru complexes

The synthesised Ru complexes, $[Ru(dmbpy)_3](PF_6)_2$ and $[Ru(dmo\text{-}bpy)_3](PF_6)_2$, were characterised by NMR and HRMS. The raw NMR spectra obtained from the Ru complexes are shown in Figure S4. HRMS revealed that the average molecular weight of $[Ru(dmbpy)_3]^{2+}$ and $[Ru(dmo\text{-}bpy)_3]^{2+}$ was 653.7777 and 749.7741 g mol^{-1}, respectively.

$[Ru(dmbpy)_3](PF_6)_2$: ^1H NMR (300 MHz, d-methanol) δ 8.53 (s, 6H), 7.60 (d, J = 5.76 Hz, 6H), 7.29 (d, J = 6.99 Hz, 6H), 2.56 (s, 18H); HRMS (ESI-TOF) m/z: calcd for $C_{36}H_{36}N_6Ru$ $[M]^{2+}$ 327.1027, found 327.1021.

$[Ru(dmo\text{-}bpy)_3](PF_6)_2$: ^1H NMR (300 MHz, DMSO) δ 8.44 (s, 6H), 7.51 (d, J = 6.61 Hz, 6H), 7.13 (dd, J = 3.87, 6.61 Hz, 6H), 3.98 (s, 18H); HRMS (ESI-TOF) m/z: calcd for $C_{36}H_{36}N_6O_6Ru$ $[M]^{2+}$ 375.0865, found 375.0869.

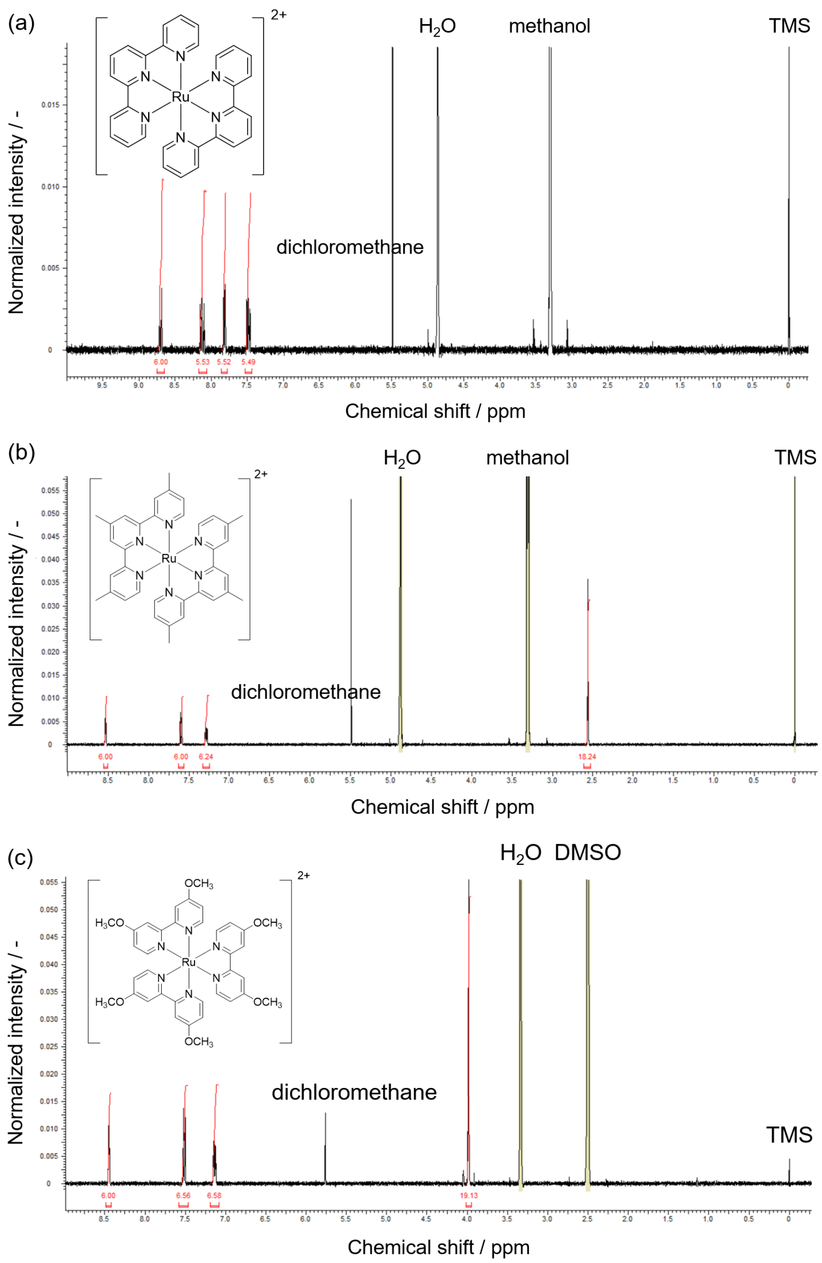

Figure S4. NMR spectra obtained from (a) $[Ru(bpy)_3](PF_6)_2$, (b) $[Ru(dmbpy)_3](PF_6)_2$, and (c) $[Ru(dmo\text{-}bpy)_3](PF_6)_2$.

Diffusion coefficients for Ru complexes

Hydrodynamic voltammograms obtained using the Pt RDE in electrolytes containing various Ru^{2+} complexes are presented in Figure S5. The diffusion-limited current was proportional to the square root of the rotation speed according to the following Levich equation, where i_{diff}, n, F, D_{RDE}, ω, v, and C represent the diffusion-limited current density, the number of electrons involved in the reaction, Faraday's constant, the diffusion coefficient for the reactant determined by Levich plots, the rotation speed of the disk electrode, the kinematic viscosity of the electrolyte, and the bulk concentration of the reactant, respectively.[7]

$$i_{diff} = 0.62nFD_{RDE}^{2/3}\omega^{1/2}v^{-1/6}C \qquad \text{(S1)}$$

From the slopes of the Levich plots, the diffusion coefficients for $[Ru(bpy)_3]^{2+}$, $[Ru(dmbpy)_3]^{2+}$, and $[Ru(dmo\text{-}bpy)_3]^{2+}$ were 9.92×10^{-6}, 9.92×10^{-6}, and 1.01×10^{-5} $cm^2\ s^{-1}$, respectively. CVs obtained using the Pt disk electrode showed that the peak anodic current was proportional to the square root of the scan rate for the electrode potential (Figure S6) according to the following Randles−Sevcik equation, where i_{pa}, v, and D_{CV} represent the peak anodic current in the CV, the scan rate for the electrode potential, and the diffusion coefficient for the reactant determined by changing the scan rate during CV measurements, respectively.[8]

$$i_{pa} = 2.69 \times 10^5 \times n^{3/2}Cv^{1/2}D_{CV}^{1/2} \qquad \text{(S2)}$$

The diffusion coefficients determined from the CVs measured by varying the scan rate were calculated to be 1.08×10^{-5}, 1.07×10^{-5}, and 1.12×10^{-5} $cm^2\ s^{-1}$ for $[Ru(bpy)_3]^{2+}$, $[Ru(dmbpy)_3]^{2+}$, and $[Ru(dmo\text{-}bpy)_3]^{2+}$, respectively. Thus, similar diffusion coefficients were obtained using the different methods.

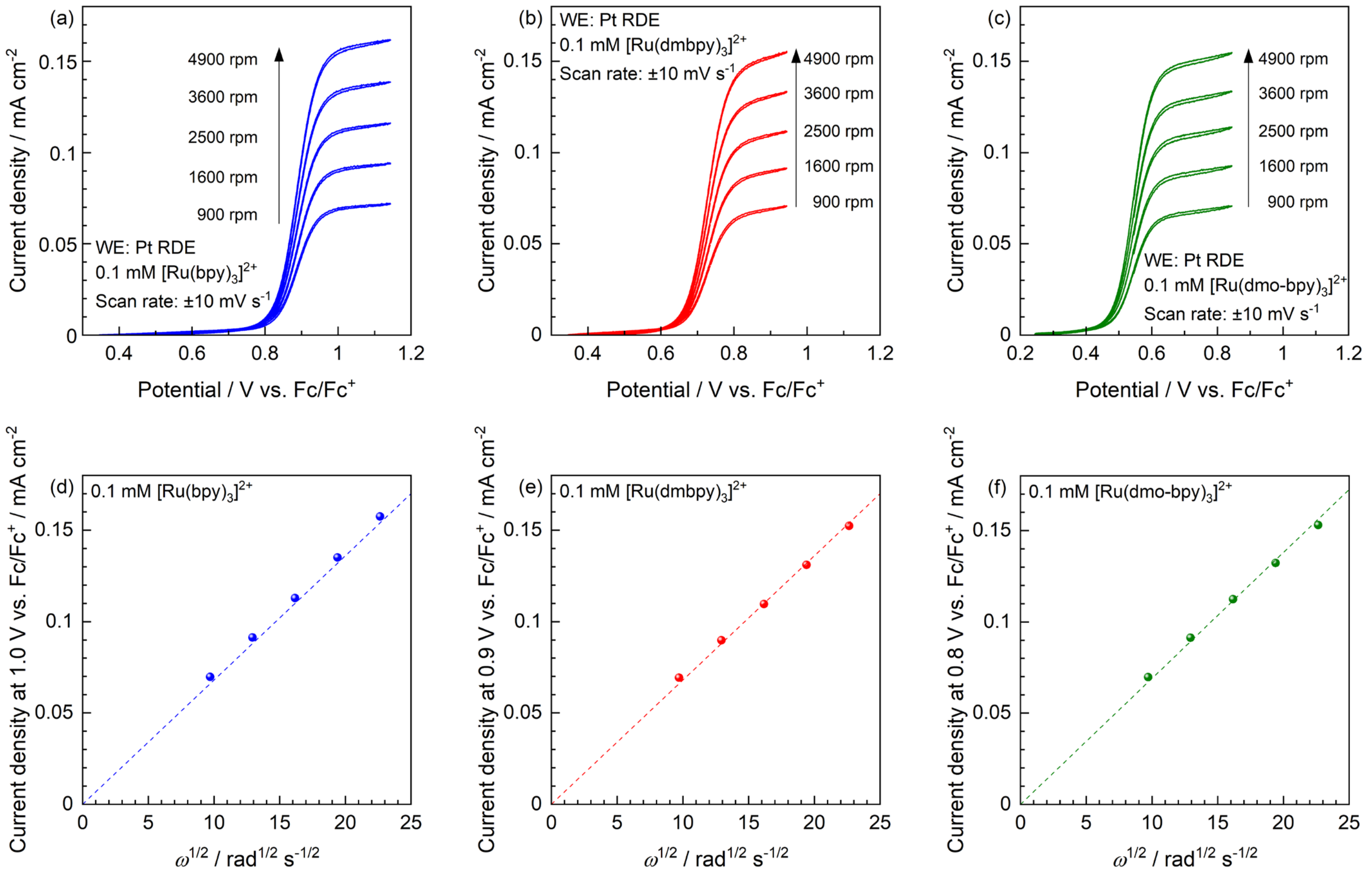

Figure S5. Current-potential curves obtained using Pt RDE with various rotation speeds in acetonitrile electrolyte containing 0.1 M $TBAPF_6$ and 0.1 mM (a) $[Ru(bpy)_3]^{2+}$, (b) $[Ru(dmbpy)_3]^{2+}$, and (c) $[Ru(dmo\text{-}bpy)_3]^{2+}$. (d–f) corresponding Levich plots.

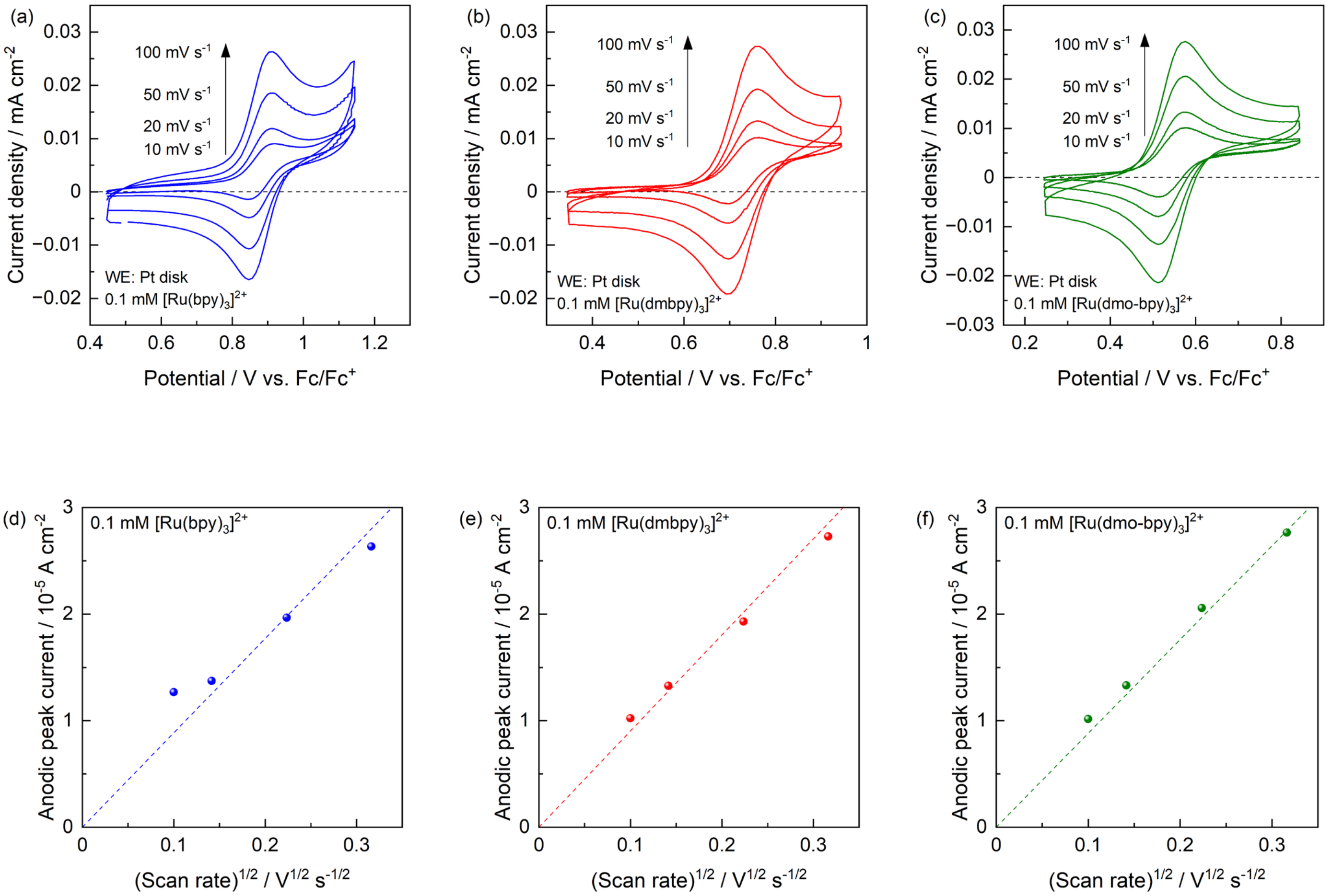

Figure S6. CVs obtained using Pt disk electrode with various potential scan rates in acetonitrile electrolyte containing 0.1 M $TBAPF_6$ and 0.1 mM (a) $[Ru(bpy)_3]^{2+}$, (b) $[Ru(dmbpy)_3]^{2+}$, and (c) $[Ru(dmo\text{-}bpy)_3]^{2+}$. (d–f) Peak anodic current as function of square root of scan rate.

Characterisation of $Zn_{0.25}Cd_{0.75}Se$ photocatalyst particles

The synthesised $Zn_{0.25}Cd_{0.75}Se$ particles had irregular shapes and a relatively wide size distribution ranging from submicron- to micron-order.[1] In literature reports, the assembly of photocatalyst particles anchored on the metal layer prepared by the PT method is typically fixed on the secondary glass substrate using double-sided carbon tape. However, in an acetonitrile environment, there is a possibility of deterioration of the carbon tape. Additionally, using the conventional fixing method, redox reactions of the Ru complexes also occur at the exposed backside metal layer between neighboring photocatalyst particles (Figure S7a). To prevent the generation of an undesired dark current associated with this, the photoanodes prepared by the PT method were fixed on the secondary glass substrate using an organic-solvent-resistant epoxy resin. The resin penetrates the metal layer and fills the voids between the photocatalyst particles (Figure S7b). Consequently, only semiconductor particles were exposed to the electrolyte.[6]

The $Zn_{0.25}Cd_{0.75}Se$ particles exhibited an absorption edge at around 700 nm, and the XRD pattern was assigned to a wurtzite-type structure (Figure S8), consistent with previous reports.[1] The PEC performance of the $Zn_{0.25}Cd_{0.75}Se$ photoanode was evaluated in acetonitrile electrolytes containing various Ru complexes with Ru^{2+}:Ru^{3+} molar ratios of unity under illumination by simulated sunlight (Figure S9). The photocurrent generated by the photoanode combined with $[Ru(bpy)_3]^{3+/2+}$ or $[Ru(dmbpy)_3]^{3+/2+}$ was larger than that for $[Ru(dmo\text{-}bpy)_3]^{3+/2+}$ over the entire potential range (Figure S9a), which is consistent with the results obtained using 600-nm monochromatic light described in the main manuscript. However, the photocurrent gradually decreased over time irrespective of the type of Ru complex, possibly due to deterioration of the electrolyte as a result of light absorption by the Ru complex, as described later.

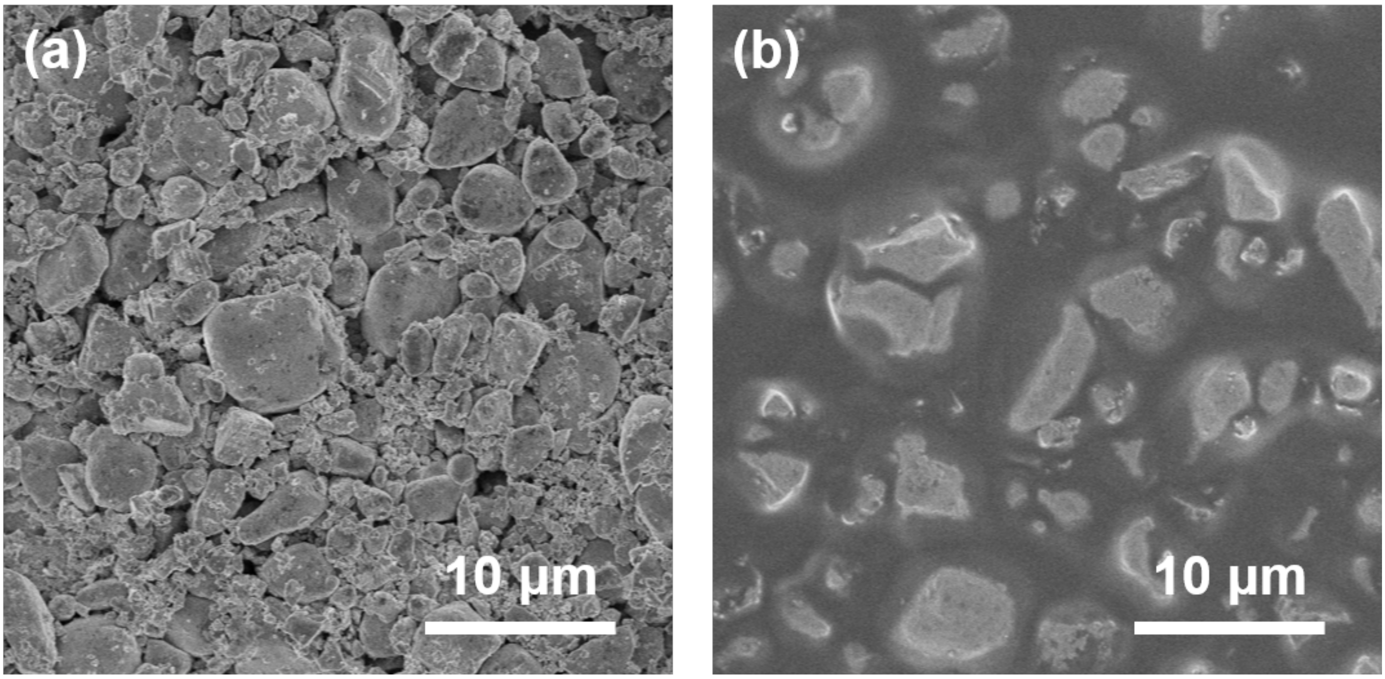

Figure S7. Top-view SEM images of particulate $Zn_{0.25}Cd_{0.75}Se$ photoanode fixed on secondary glass substrate by (a) conventional double-sided carbon tape or (b) organic-solvent-resistant epoxy resin. (a) Exposed backside Ti layer at voids between photocatalyst particles. (b) Voids between particles filled with insulating epoxy resin.

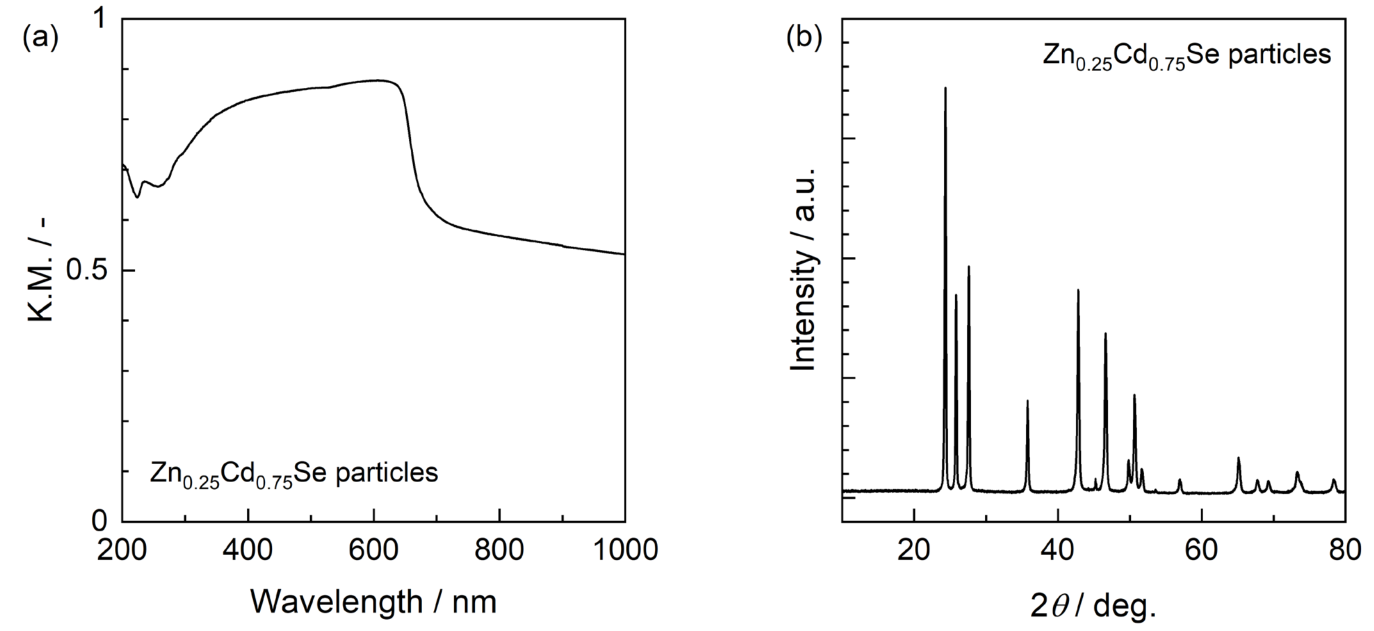

Figure S8. (a) DR spectrum and (b) XRD pattern for $Zn_{0.25}Cd_{0.75}Se$ particles.

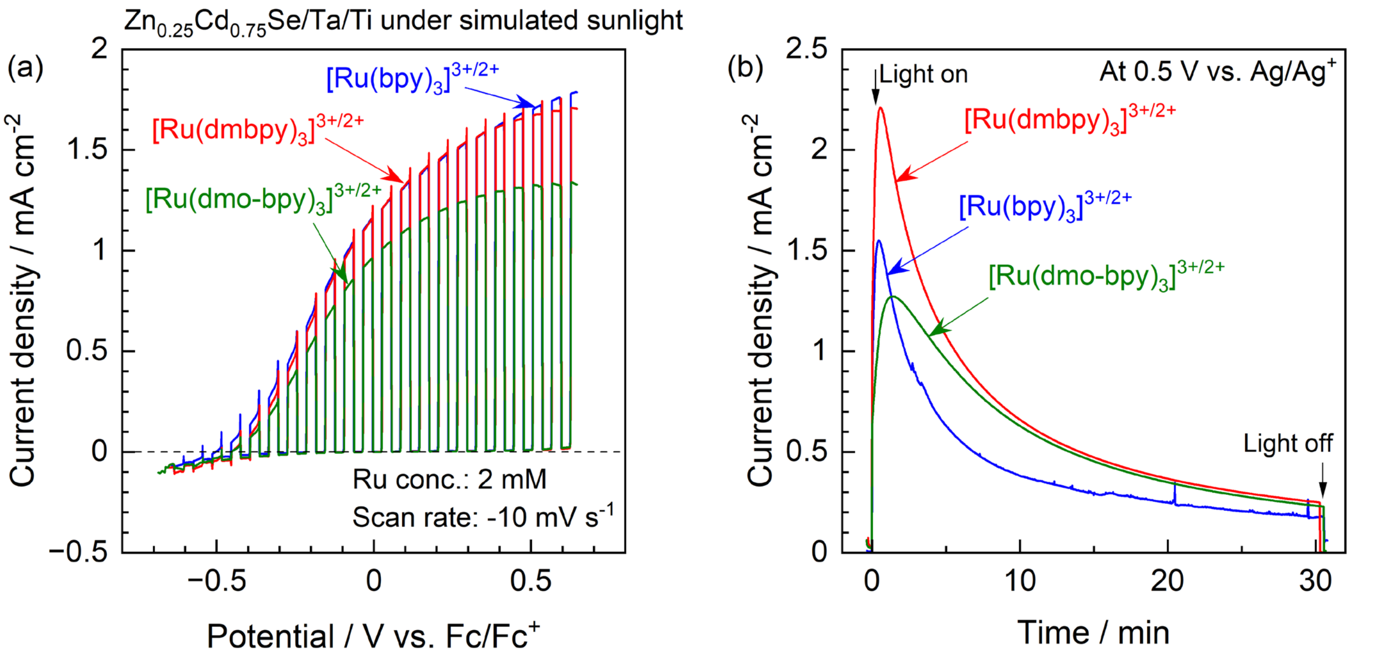

Figure S9. (a) Current-potential curves and (b) current-time curves for $Zn_{0.25}Cd_{0.75}Se$ photoanode in acetonitrile electrolyte containing equimolar amounts of Ru^{2+} and Ru^{3+} complexes with total concentration of 2 mM and 0.1 M $TBAPF_6$ under illumination by simulated sunlight.

Absorption spectra of acetonitrile electrolyte containing Ru complexes

Absorption spectra of acetonitrile electrolytes containing the Ru complexes are shown in Figure S10. The absorption peak is located at a wavelength of 450, 459, and 477 nm for $[Ru(bpy)_3]^{2+}$, $[Ru(dmbpy)_3]^{2+}$, and $[Ru(dmo\text{-}bpy)_3]^{2+}$, respectively. This strong absorption is assigned to metal-to-ligand charge transfer (MLCT) in the Ru complexes.[9, 10] For an electrolyte electrolysed at +0.2 V relative to the half-wave potential ($E_{1/2}$) for the Ru complex, the Ru^{2+}:Ru^{3+} molar ratio in the electrolyte should be almost 0:1 based on the Nernst equation. The main absorption peak at 400–500 nm assigned to MLCT in the Ru^{3+} complex is significantly lower than that for Ru^{2+}, while the Ru^{3+} complex showed an additional weak absorption peak at 600–800 nm, possibly due to ligand-to-metal charge transfer (LMCT).[11, 12] The electrolytes containing equimolar amounts of Ru^{2+} and Ru^{3+}, which were prepared by bulk electrolysis at $E_{1/2}$, showed absorption spectra that were intermediate between those for the Ru^{2+} and Ru^{3+} complexes. The absorbance at a given wavelength was almost proportional to Ru^{2+}/Ru^{3+} ratio. Thus, the Ru^{2+}/Ru^{3+} ratio in the bulk electrolyte after the PEC reaction could be estimated based on the absorption spectrum of the electrolyte, as described later.

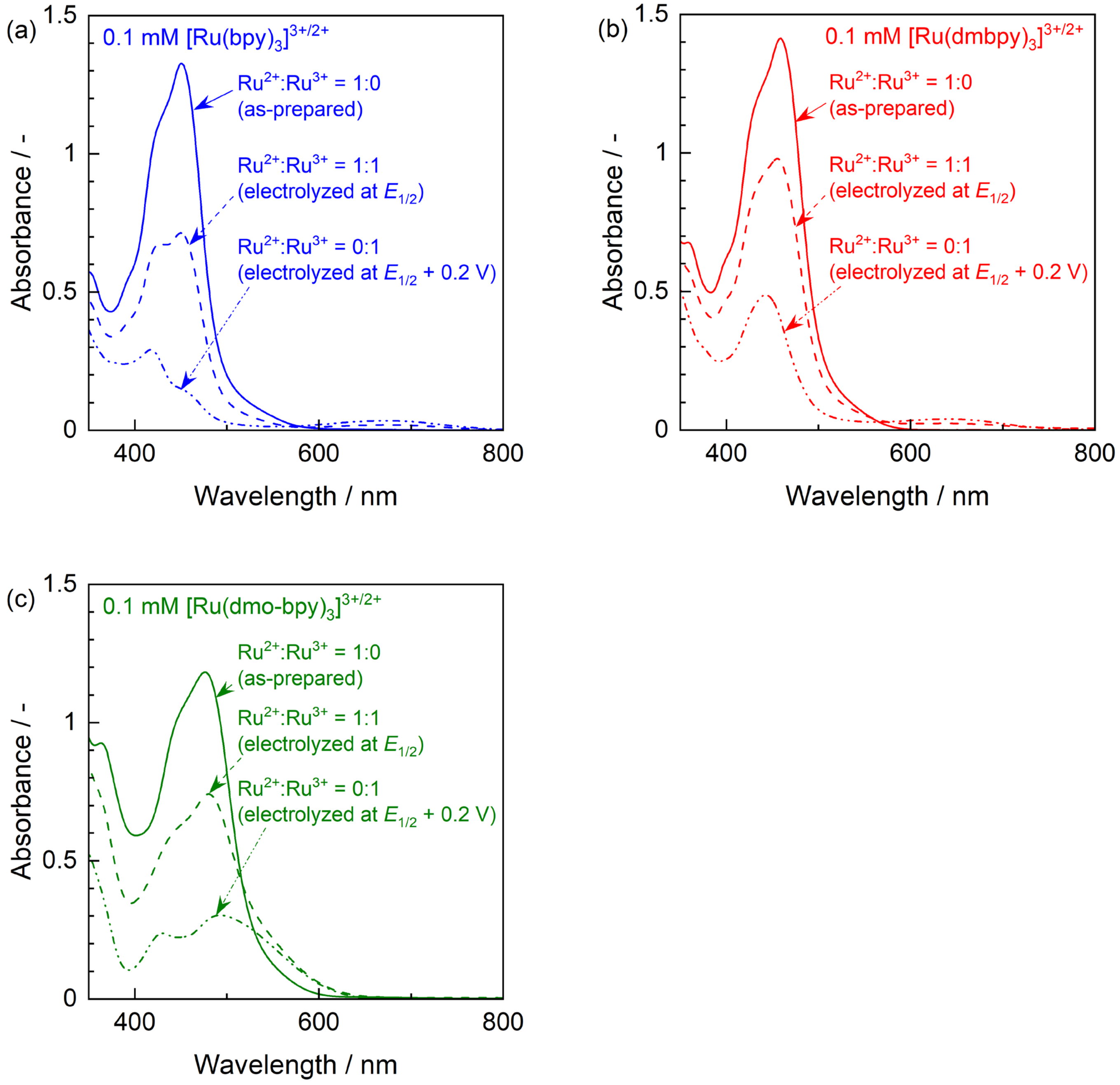

Figure S10. Absorption spectra of acetonitrile electrolyte containing (a) $[Ru(bpy)_3]^{3+/2+}$, (b) $[Ru(dmbpy)_3]^{3+/2+}$, or (c) $[Ru(dmo\text{-}bpy)_3]^{3+/2+}$.

Influence of concentration of ferrocene on PEC performance

The dependence of the IPCEs on the electrode potential for a $Zn_{0.25}Cd_{0.75}Se$ photoanode in an acetonitrile electrolyte containing 1 or 2 mM ferrocene is shown in Figure S11, together with the results obtained using $[Ru(dmo\text{-}bpy)_3]^{3+/2+}$. It was found that the IPCE was almost independent of the ferrocene concentration, consistent with the photocurrent being mainly governed by charge separation in the semiconductor. For PEC trials using the Ru redox shuttle, the nonaqueous electrolyte should contain 1 mM Ru^{2+} and 1 mM Ru^{3+} complexes after the bulk electrolysis, whereas the total concentration of the Ru redox shuttle is 2 mM. For the purpose of unifying the experimental conditions (i.e., concentration of redox reagents contained in the electrolyte), the ferrocene concentration was fixed at 2 mM in the main manuscript.

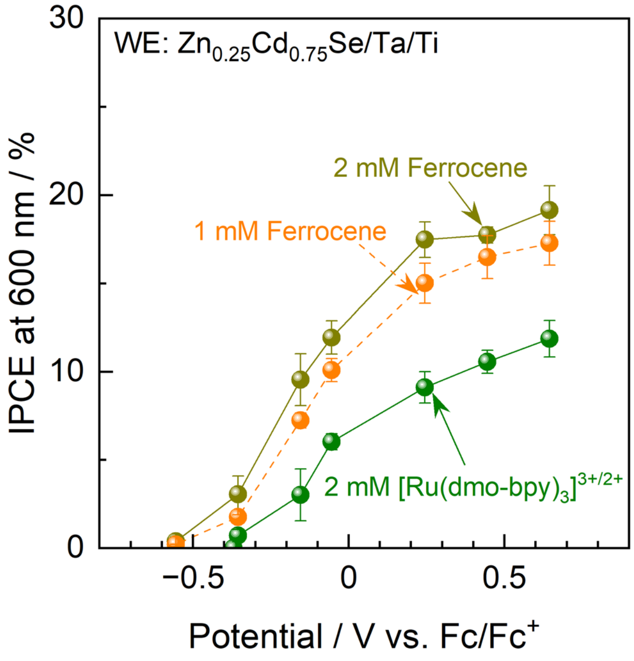

Figure S11. IPCE-potential curves for $Zn_{0.25}Cd_{0.75}Se$ photoanode under illumination by 600-nm monochromatic light emitted from Xe lamp. The acetonitrile electrolyte contained1 or 2 mM ferrocene, or 2 mM $[Ru(dmo\text{-}bpy)_3]^{3+/2+}$ (equimolar amounts of Ru^{2+} and Ru^{3+}) and 0.1 M $TBAPF_6$.

Comparison of photocurrent generated by $Zn_{0.25}Cd_{0.75}Se$ photoanodes and diffusion-limited current for redox shuttle

If the photocurrent is limited by reactant diffusion, it no longer reflects the thermodynamic aspects of the PEC reaction. Thus, we determined whether the PEC reaction was governed by the diffusion limit for the Ru complex, and the results are shown in Figure S12. Figure S12a shows a typical current-time curve for the photoanode in an acetonitrile electrolyte containing a Ru complex under illumination by 600-nm monochromatic light, which was used to calculate the IPCE presented in the main manuscript. Figure S12b shows the current-potential curve for a Pt disk electrode in a nonaqueous electrolyte under vigorous stirring. The maximum photocurrent due to oxidation of Ru^{2+} by photogenerated holes in the present study was clearly smaller than the diffusion-limited current in an electrolyte stirred by a magnetic stirring bar. Therefore, it can be concluded that the photocurrent observed in this study was governed by charge transfer, rather than by diffusion in the electrolyte.

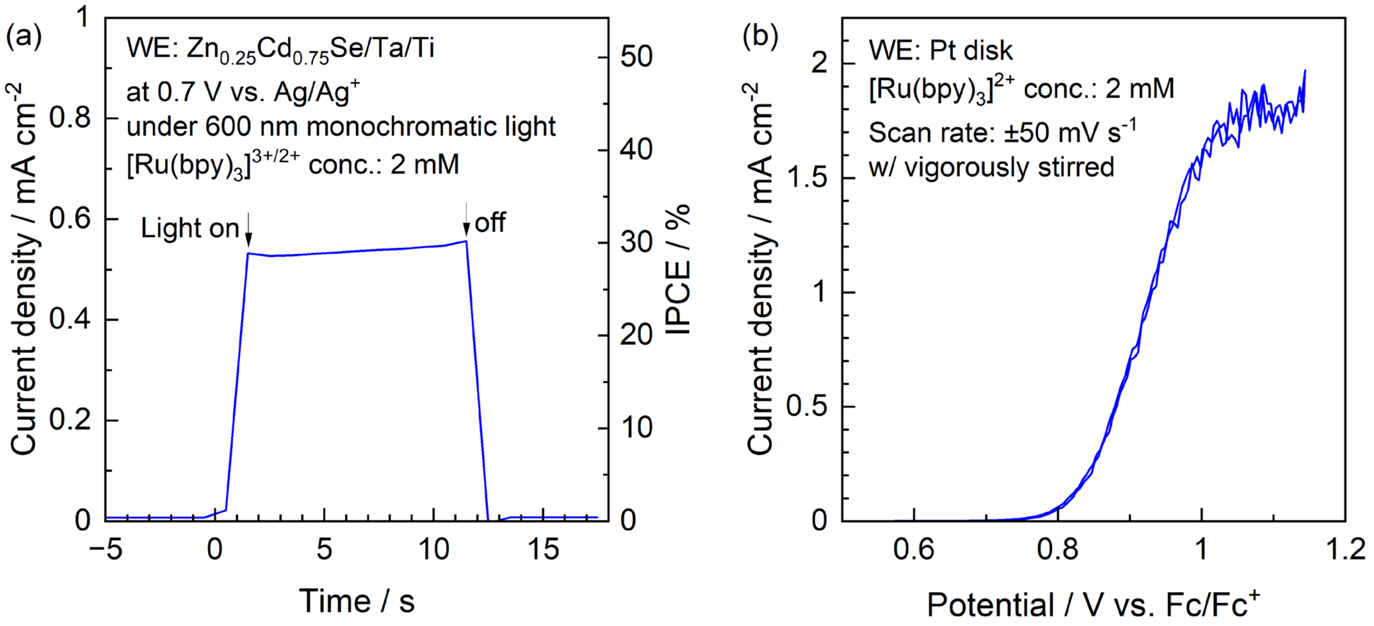

Figure S12. (a) Current-time curve for $Zn_{0.25}Cd_{0.75}Se$ photoanode at 0.7 V vs. Ag/Ag^{+} under illumination by 600-nm monochromatic light from Xe lamp and (b) current-potential curve for Pt disk electrode. The working electrodes were immersed in an acetonitrile electrolyte containing the Ru complex, which was stirred vigorously.

Change in morphology and composition of $Zn_{0.25}Cd_{0.75}Se$ photoanodes induced by photocorrosion

Figure S13 shows cross-sectional SEM images of $Zn_{0.25}Cd_{0.75}Se$ photoanodes before and after PEC measurements. Almost one monolayer of photocatalyst particles were firmly anchored to the backside metal electrode using the PT method (Figure S13a). After the PEC measurement using $[Ru(bpy)_3]^{3+/2+}$, the surface of the photocatalyst particles was covered with a sponge-like layer containing many voids (Figure S13b). However, the appearance of the photocatalyst particles was almost unchanged after the PEC measurements using $[Ru(dmo\text{-}bpy)_3]^{3+/2+}$ (Figure S13c). EDS line analyses further revealed that the photocatalyst surface after the PEC trial using $[Ru(bpy)_3]^{3+/2+}$ contained much more Se and less cation species than the pristine photoanode (Figures S13d and S13e). This is consistent with competition between PEC oxidation of $[Ru(bpy)_3]^{2+}$ and photocorrosion, whereas little photocorrosion occurred when $[Ru(dmo\text{-}bpy)_3]^{3+/2+}$ was employed, as discussed in the main manuscript. The thickness of the photocorroded layer after the PEC reaction using $[Ru(bpy)_3]^{3+/2+}$ can be roughly estimated to be on the order of microns (Figures S13b and S13e).

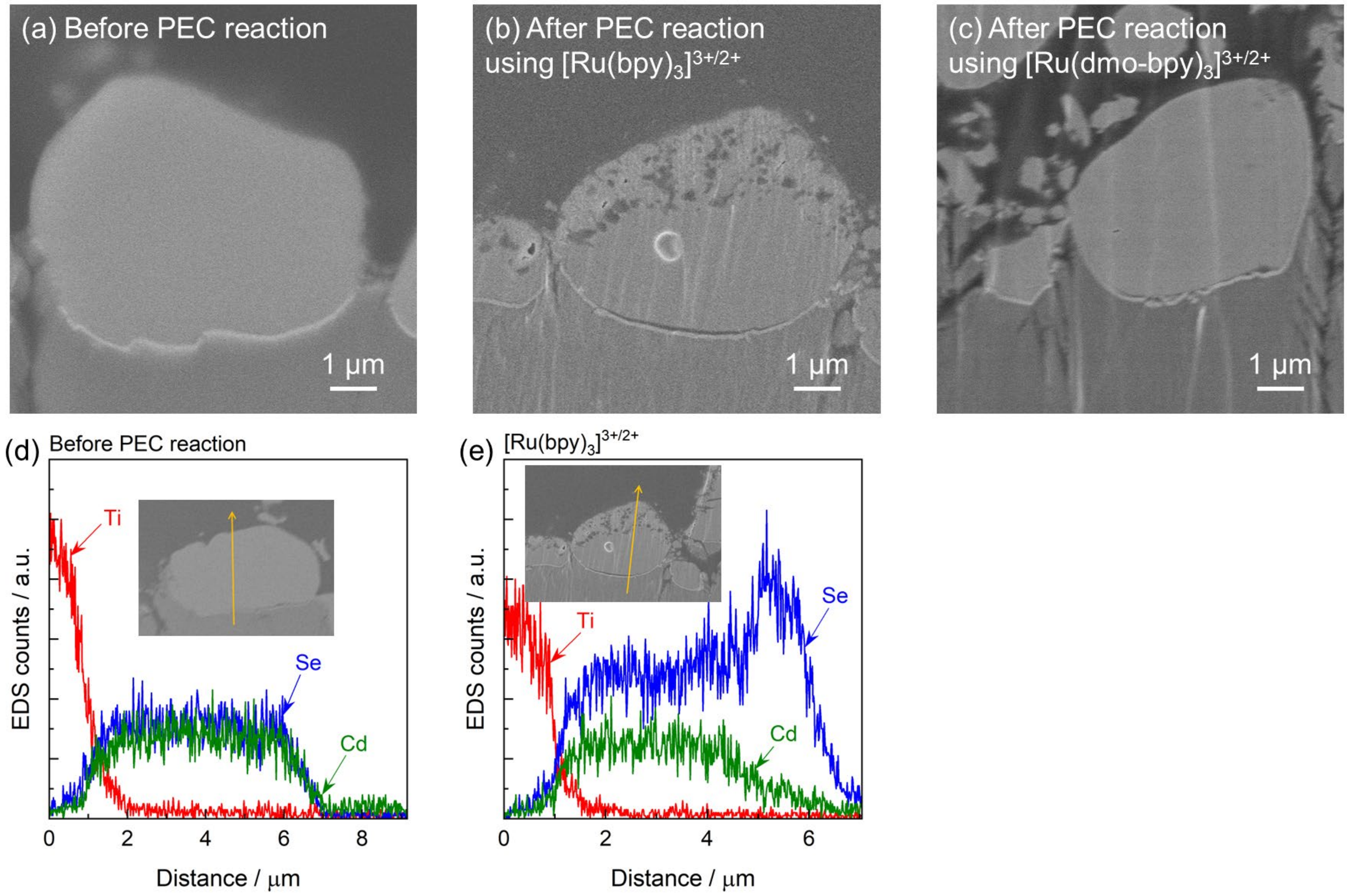

Figure S13. Cross-sectional SEM images of $Zn_{0.25}Cd_{0.75}Se$ photoanodes (a) before PEC reactions, and after PEC trials using (b) $[Ru(bpy)_3]^{3+/2+}$ and (c) $[Ru(dmo\text{-}bpy)_3]^{3+/2+}$. EDS line analyses for photoanode (d) before and (e) after PEC measurement using $[Ru(bpy)_3]^{3+/2+}$.

Ratio of Ru^{2+} to Ru^{3+} in bulk electrolyte during PEC reaction

The absorption spectra of the acetonitrile electrolyte containing the Ru complexes with various Ru^{2+}/Ru^{3+} molar ratios (Figure S10) showed that the absorbance at a given wavelength was almost proportional to the Ru^{2+}/Ru^{3+} ratio. Thus, the Ru^{2+}/Ru^{3+} ratio in the bulk electrolyte after the PEC reaction can be estimated from the absorption spectrum of the electrolyte using the calibration curves presented in Figures S14a–S14c. Time courses of the Ru^{2+}/Ru^{3+} molar ratio during the PEC reaction are shown in Figures S14d–S14f. When the electrolyte was irradiated with simulated sunlight, the Ru^{2+} content gradually increased irrespective of the type of Ru complex used. A typical example using $[Ru(bpy)_3]^{3+/2+}$ is given in Figure S14d. This implies photo-induced self-reduction of Ru^{3+} into Ru^{2+} species. When the electrolyte was irradiated with 600-nm monochromatic light, the Ru^{2+}/Ru^{3+} molar ratios were almost unchanged due to the relatively low absorption coefficients for the Ru complexes at this wavelength. Because the counter-electrode (cathode) always causes reduction of Ru^{3+} species during

the PEC reaction, a significant amount of photocorrosion would be expected to lead to an increase in the Ru^{2+} content in the electrolyte. However, interestingly, the electrolyte in which the $Zn_{0.25}Cd_{0.75}Se$ photoanode performed the PEC reaction also showed constant Ru^{2+}/Ru^{3+} molar ratios, even though the $[Ru(bpy)_3]^{3+/2+}$ and $[Ru(dmbpy)_3]^{3+/2+}$ caused serious photocorrosion that competed with PEC oxidation of Ru^{2+}. This is thought to be because photocorrosion occurred only at the surface and thus the amount of eluted $Zn_{0.25}Cd_{0.75}Se$ was much smaller than the total number of moles of Ru complex contained in the electrolyte.

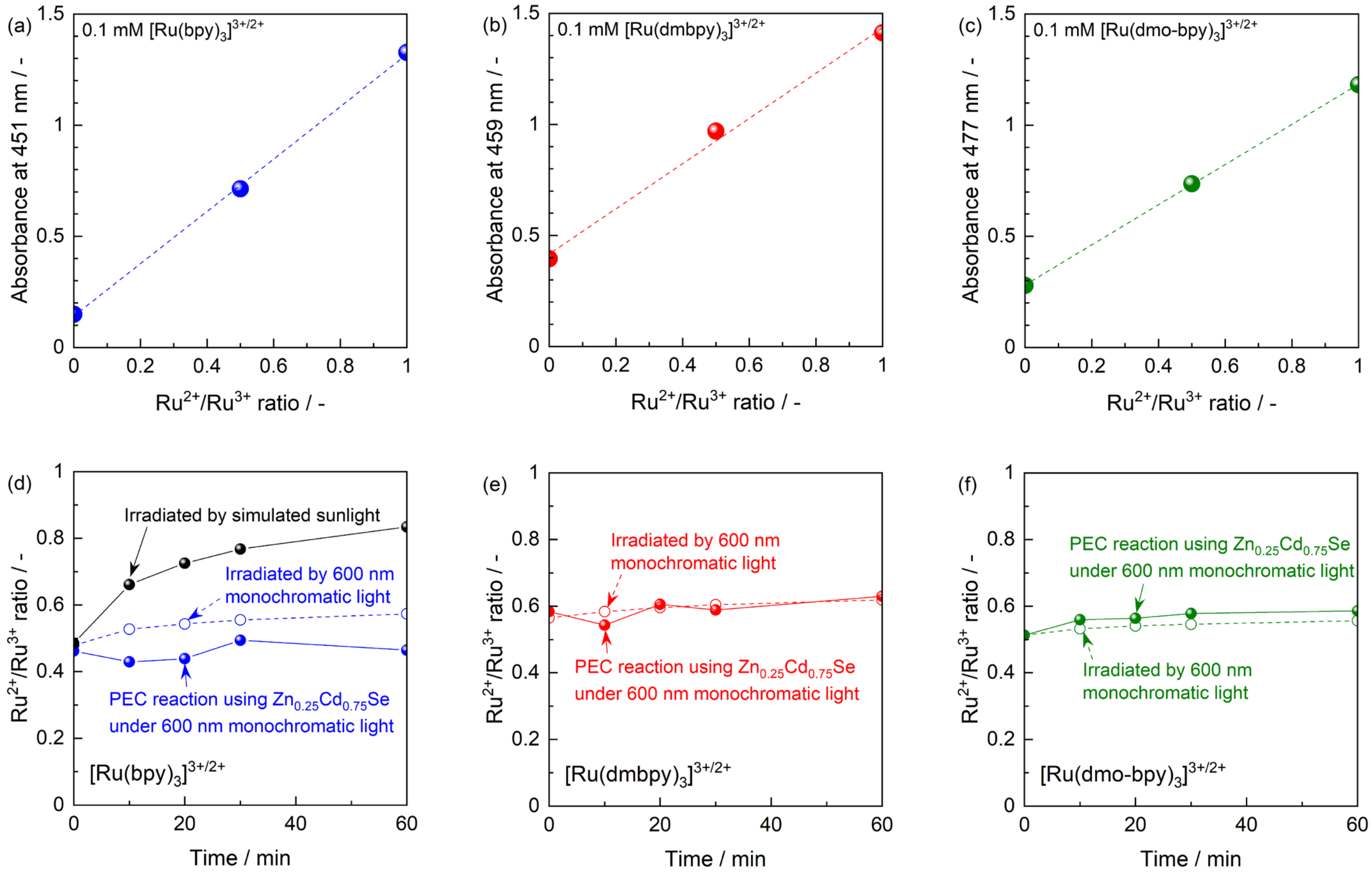

Figure S14. Absorbance of (a) $[Ru(bpy)_3]^{3+/2+}$ at 451 nm, (b) $[Ru(dmbpy)_3]^{3+/2+}$ at 459 nm, and (c) $[Ru(dmo\text{-}bpy)_3]^{3+/2+}$ at 477 nm as functions of Ru^{2+}/Ru^{3+} molar ratio. Time courses of Ru^{2+}/Ru^{3+} molar ratio under irradiation by 600-nm monochromatic light and during PEC reaction using $Zn_{0.25}Cd_{0.75}Se$ photoanodes employing (d) $[Ru(bpy)_3]^{3+/2+}$, (e) $[Ru(dmbpy)_3]^{3+/2+}$, and (f) $[Ru(dmo\text{-}bpy)_3]^{3+/2+}$ as redox shuttle. The time course of the $[Ru(bpy)_3]^{3+/2+}$ molar ratio under illumination by simulated sunlight is also provided in d.

Maximum expected degree of photocorrosion

From the XPS depth profile (Figure 4 in the main manuscript), the elemental composition at a depth of approximately 100 nm from the surface of $Zn_{0.25}Cd_{0.75}Se$ combined with $[Ru(dmbpy)_3]^{3+/2+}$ was almost stoichiometric. Meanwhile, almost all regions within this depth for $Zn_{0.25}Cd_{0.75}Se$ combined with $[Ru(bpy)_3]^{3+/2+}$ seemed completely photocorroded. Here, we roughly evaluated the eluted amounts of $Zn_{0.25}Cd_{0.75}Se$ if all the photocatalyst surface within a 100-nm depth was photocorroded, as summarised in Figure S15. Based on the SEM observations, the average diameter of $Zn_{0.25}Cd_{0.75}Se$ particles was 5.2 μm. Assuming that half-spheres with a radius of 2.6 μm densely covered the photoelectrode surface, the number of half-spheres per geometric area of the photoelectrode was 4.7×10^6 cm^{-2}. Assuming that the photocatalyst near-surface region within a depth of 100 nm was photocorroded, the volumes of the corroded and non-corroded parts of the photocatalysts per particle were 4.1×10^9 nm^3 and 3.3×10^{10} nm^3, respectively. Considering that the cell volume of $Zn_{0.25}Cd_{0.75}Se$ is 0.319 nm^3 [1] and that the unit cell contains four pairs, the photocorroded amount of $Zn_{0.25}Cd_{0.75}Se$ per geometric area of the photoelectrode can be calculated as 0.4 μmol cm^{-2}.

The total charge that passed during the chronoamperometry (CA) measurements shown in Figure 4a in the main manuscript was 0.3–0.4 μC cm^{-2}. Considering that the photocorrosion reaction of $Zn_{0.25}Cd_{0.75}Se$ proceeds via a two-electron process, the faradaic efficiency for photocorrosion is expected to be 18–24% at most. It should be noted that photocorrosion barely occurred during PEC oxidation of $[Ru(dmo\text{-}bpy)_3]^{2+}$, and that the photocorrosion that competed with PEC oxidation

of [Ru(dmbpy)$_3$]$^{2+}$ proceeded only to a depth of 100 nm. Therefore, the actual faradaic efficiency for photocorrosion that competes with PEC oxidation of [Ru(dmo-bpy)$_3$]$^{2+}$ and [Ru(dmbpy)$_3$]$^{2+}$ should be much smaller than the above expectation.

During the PEC measurements, 15 mL of the nonaqueous electrolyte containing 2 mM Ru complexes in total was typically used. Thus, the electrolyte contained 30 μmol of Ru complexes. Because the geometric surface area of the typical photoelectrodes used was 0.12–0.19 cm^2, the eluted amounts of $Zn_{0.25}Cd_{0.75}Se$ were expected to be only 0.3–0.5% of the Ru complexes contained in the electrolyte at most. Therefore, it can be considered that, even if all the photocatalyst surface to a depth of 100 nm was photocorroded, the Ru^{2+}/Ru^{3+} ratio in the bulk electrolyte should be almost unchanged. This point is consistent to discussion in the previous section based on the absorption spectra of the electrolyte before and after the PEC reaction. The calculated values for each Ru complex are summarised in Table S1. Furthermore, even assuming that the photocatalyst surface was photocorroded to a depth of 1 μm after the PEC reaction using [Ru(bpy)$_3$]$^{3+/2+}$, although the faradaic efficiency for photocorrosion should be 100%, the amount of eluted photocatalytic material should still be only 2–4% of the total number of moles of Ru complex.

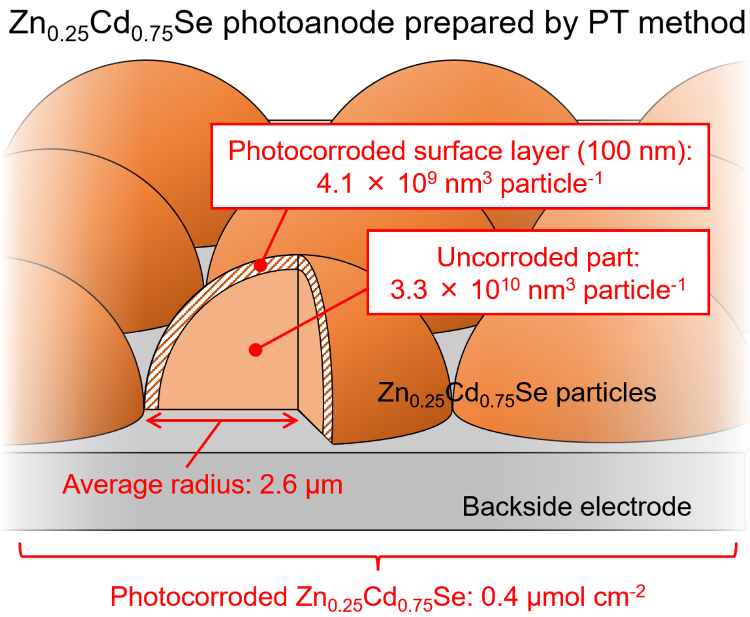

Figure S15. Calculation of eluted number of moles of $Zn_{0.25}Cd_{0.75}Se$ assuming that all photocatalyst surface within depth of 100 nm was photocorroded.

Table S1. Maximum expected degree of photocorrosion and calculation parameters.

	Geometric surface area / cm^2 [a]	Maximum photocorroded $Zn_{0.25}Cd_{0.75}Se$ / μmol[b]	Total charge passed during the CA measurements / μC cm^{-2}	Mole number of electrons passed during the CA measurements / μmol	Maximum faradaic efficiency for the photocorrosion / %	Molar ratio of eluted cations to Ru complexes contained in the electrolyte / %
[Ru(dmo-bpy)$_3$]$^{3+/2+}$	0.128	5.13×10^{-2}	0.317	0.420	24.4	0.342
[Ru(dmbpy)$_3$]$^{3+/2+}$	0.187	7.49×10^{-2}	0.434	0.840	17.8	0.500
[Ru(bpy)$_3$]$^{3+/2+}$	0.115	4.61×10^{-2}	0.433	0.516	17.9	0.307

[a] Geometric surface areas of specimens used for experiments described in Figure 4 in main manuscript. [b] Assuming 0.4 μmol cm^{-2}.

XPS analysis of $Zn_{0.25}Cd_{0.75}Se$ photoanodes

A typical XPS spectrum obtained from a $Zn_{0.25}Cd_{0.75}Se$ photoanode is shown in Figure S16a. The peak positions assigned to Zn 2p (Figure S16b) and Cd 3d (Figure S16c) were slightly shifted to higher binding energies after the PEC reaction employing a $[Ru(bpy)_3]^{3+/2+}$ or $[Ru(dmbpy)_3]^{3+/2+}$ redox shuttle. The Se 3d XPS spectra obtained from the photoanodes combined with $[Ru(bpy)_3]^{3+/2+}$ or $[Ru(dmbpy)_3]^{3+/2+}$ also shifted to higher binding energies (Figure S16d), implying the metallic nature of the surface Se. Only for the case of the $[Ru(dmo\text{-}bpy)_3]^{3+/2+}$ redox shuttle, the photoanode generated almost identical XPS signals to those for the as-prepared specimen. These observations suggest the resistance of the photoanode to photocorrosion only when employing $[Ru(dmo\text{-}bpy)_3]^{3+/2+}$.

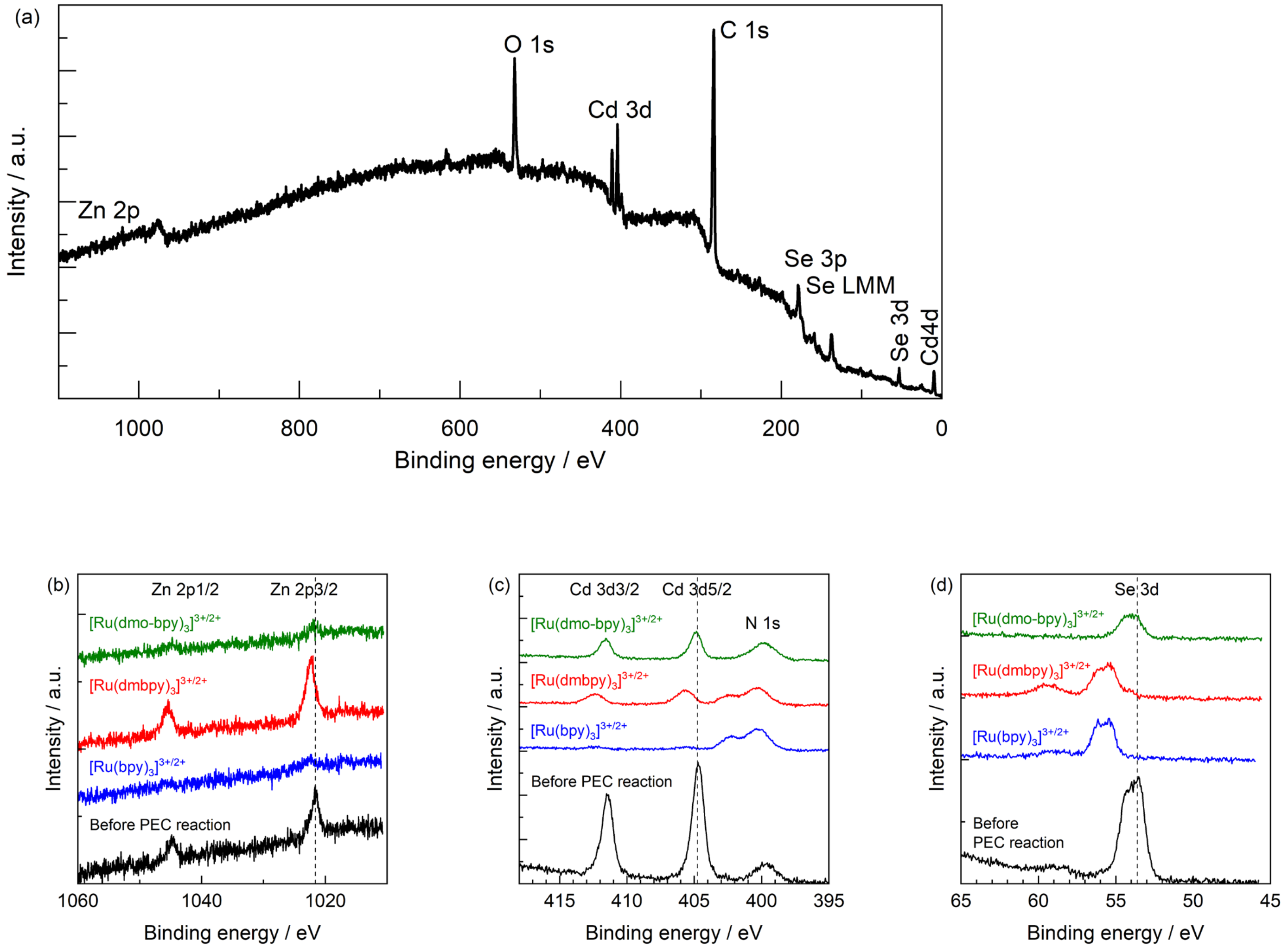

Figure S16. (a) Typical XPS spectrum obtained from $Zn_{0.25}Cd_{0.75}Se$ photoanode. (b) Zn 2p, (c) Cd 3d, and (d) Se 3d XPS signals obtained from $Zn_{0.25}Cd_{0.75}Se$ photoanodes before and after PEC reaction employing $[Ru(bpy)_3]^{3+/2+}$, $[Ru(dmbpy)_3]^{3+/2+}$, and $[Ru(dmo\text{-}bpy)_3]^{3+/2+}$ as redox shuttle.

Change in IPCE-potential curves after PEC reaction

The electrode potential dependence of the IPCE for the $Zn_{0.25}Cd_{0.75}Se$ photoanodes after the PEC reaction under 0.5 V vs. Ag/Ag^{+} for 10 min is shown in Figure S17. For the as-prepared photoanodes (before long-term PEC reaction), $[Ru(bpy)_3]^{3+/2+}$ or $[Ru(dmbpy)_3]^{3+/2+}$ provided the highest photocurrent among the present redox species, as discussed in the main manuscript (Figure 3). Meanwhile, after a long-term PEC reaction, the IPCE for the photoanode in the electrolyte containing $[Ru(bpy)_3]^{3+/2+}$ or $[Ru(dmbpy)_3]^{3+/2+}$ drastically decreased across the entire potential range, while the IPCE-potential profiles were almost unchanged for the case of $[Ru(dmo\text{-}bpy)_3]^{3+/2+}$ or ferrocene (Figure S17). Consequently, the photoanode generated an almost identical photocurrent irrespective of the equilibrium potential of the redox as far as the Ru complexes were employed, and the photocurrent originating from oxidation of ferrocene was still larger than that for the Ru complexes.

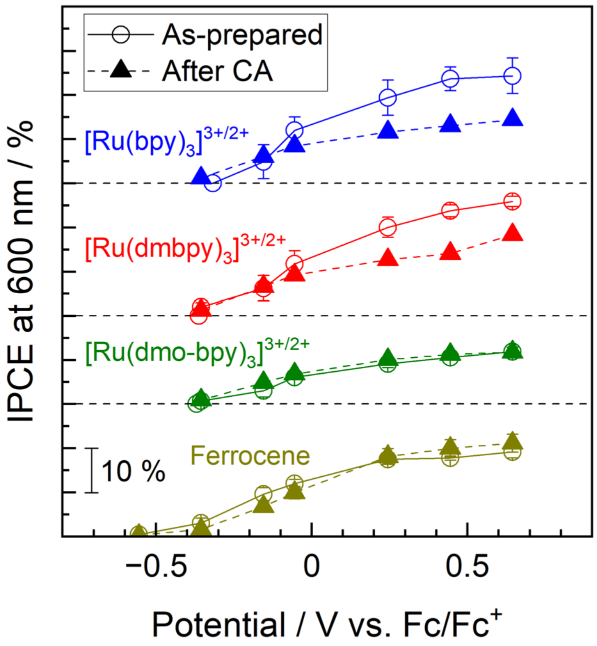

Figure S17. IPCE-potential curves for $Zn_{0.25}Cd_{0.75}Se$ photoanode before and after long-term PEC reaction under 0.5 V vs. Ag/Ag^{+} for 10 min. The photoanodes were illuminated by 600-nm monochromatic light emitted from a Xe lamp. The acetonitrile electrolyte contained equimolar amounts of Ru^{2+} and Ru^{3+} complexes with a total concentration of 2 mM and 0.1 M $TBAPF_6$.

PEC measurements using sacrificial reagents

PEC measurements using nonaqueous electrolyte containing methanol or triethanolamine as typical hole scavengers were performed as summarised in Figure S18. Here, difference between current density values under light illumination and under dark condition was expressed as *photocurrent density* in Figures S18b and S19b. As shown in the CVs obtained by a Pt disk electrode (Figure S18a), the present nonaqueous electrolyte without a hole scavenger (that is, the acetonitrile solution containing only a supporting electrolyte) was inert at relatively wide range of potential. When methanol or triethanolamine was added to the electrolyte, significant anodic current originating from decomposition of the sacrificial reagent on Pt was observed. Electrochemical oxidation of triethanolamine proceeded at more negative potentials than the case of methanol oxidation, indicating that triethanolamine is more easily oxidised than methanol. Photocurrent generated by the $Zn_{0.25}Cd_{0.75}Se$ photoanode in the nonaqueous electrolyte without the sacrificial reagents should be fully attributable to photocorrosion (Figure S18b). PEC performance of the photoanode was improved by adding the sacrificial reagents, and triethanolamine provided much more enhanced performance than the case of methanol. This indicated that the PEC measurements using a sacrificial hole scavenger similar to the typical measurements in an aqueous condition can also be applicable to the nonaqueous condition, and that triethanolamine might be a suitable sacrificial reagent to obtain optimal possible PEC performance of this material. The difference in PEC performance between the case of triethanolamine and methanol hole scavengers should be attributable to the fact that the former is more easily oxidised than the latter. However, we could not distinguish whether this difference is attributed to thermodynamic (i.e., equilibrium potential) or kinetic aspects (i.e., number of electrons involved, rate constant, diffusion, and/or elementary steps involved) of the reaction process. Additionally, such a simple comparison between triethanolamine and methanol is incapable of assessing the existence of some recombination centres at the potential region ranging 0—1 V vs. Fc/Fc^+.

Meanwhile, PEC performance of $Zn_{0.25}Cd_{0.75}Se$ photoanode in an aqueous electrolyte containing different sacrificial hole scavengers was also evaluated as shown in Figure S19. CVs of a Pt disk electrode revealed that oxidation of sulphite and methanol proceeded at more negative potential than oxygen evolution, indicating that these sacrificial reactions are electrochemically easier than water oxidation (Figure S19a). Indeed, addition of sulphite sacrificial reagent significantly enhanced PEC performance of $Zn_{0.25}Cd_{0.75}Se$ photoanode compared to the case of the absence of hole scavengers (Figure S19b). However, addition of methanol barely affected the PEC performance, even though both sulphite and methanol have been usually used as a promising hole scavenger that should completely consume the holes arriving at the photocatalyst surface. Judging solely from these experimental results, it can be at least concluded that sulphite should be a suitable sacrificial reagent in the present case (conversely methanol was inappropriate for this photocatalytic material). Nevertheless, whether the origin of different effect of sulphite or methanol sacrificial hole scavengers on PEC performance is attributable to the thermodynamic or kinetic aspects of the reaction process can not be distinguished in principle. This is analogous to the above discussion related to the PEC measurements using sacrificial reagents in a nonaqueous environment (Figure S18).

Consequently, it can be concluded that the present nonaqueous PEC measurements using various Ru bipyridyl complexes with identical kinetic parameters but different thermodynamic equilibrium potentials as a probe should be a more suitable methodology to precisely assess some recombination centres and/or photocorrosion potential existing between the bandgap.

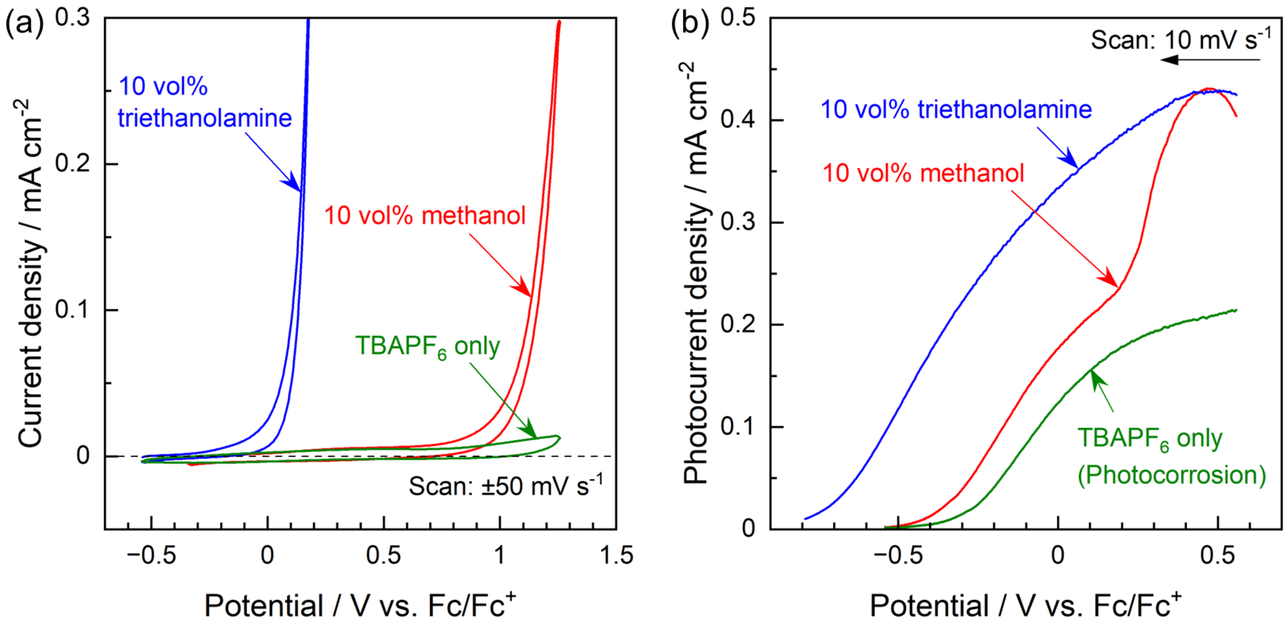

Figure S18. (a) CVs for Pt disk electrode and (b) current-potential curves of $Zn_{0.25}Cd_{0.75}Se$ photoanode in acetonitrile electrolyte with and without 10 vol% triethanolamine or methanol. During the (a) CV and (b) PEC measurements, the electrolyte was purged by Ar without and with mechanical stirring, respectively. Supporting electrolyte: 0.1 M $TBAPF_6$. Light source: 300 W Xe lamp equipped with a 600-nm monochromator.

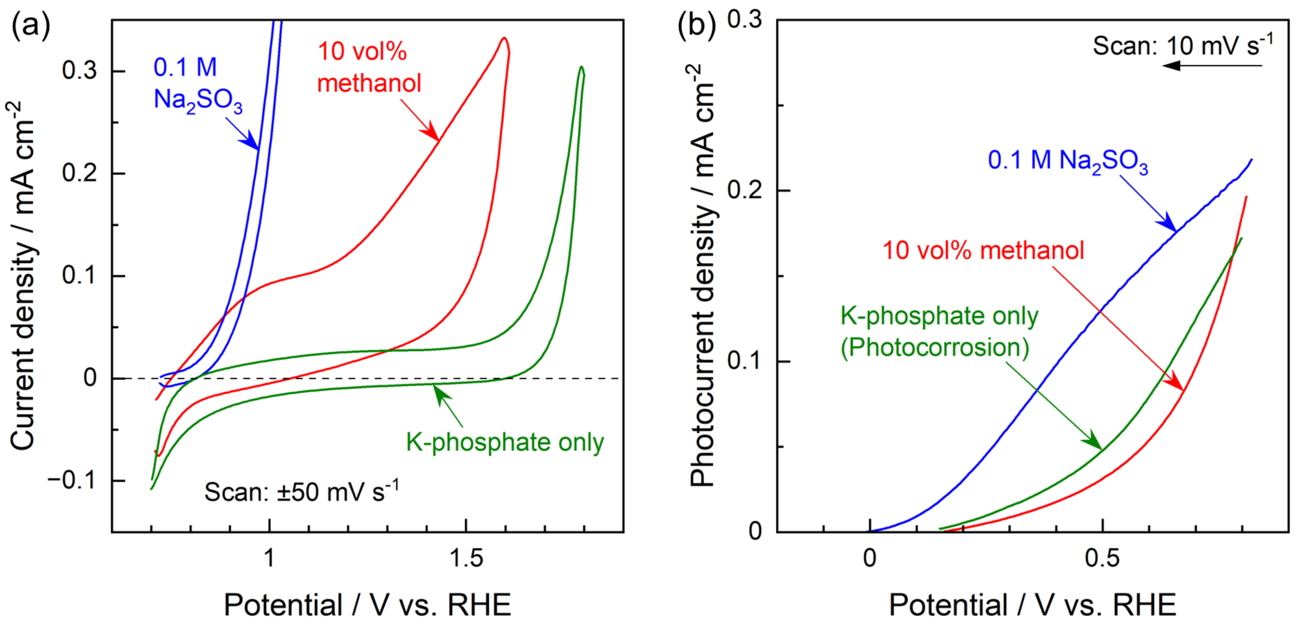

Figure S19. (a) CVs for Pt disk electrode and (b) current-potential curves of $Zn_{0.25}Cd_{0.75}Se$ photoanode in 0.1 M potassium phosphate aqueous buffered electrolyte (0.05 M KH_2PO_4/0.05 M K_2HPO_4) with and without 0.1 M Na_2SO_3 or 10 vol% methanol. During the (a) CV and (b) PEC measurements, the electrolyte was purged by Ar without and with mechanical stirring, respectively. Light source: 300 W Xe lamp equipped with a 600-nm monochromator.

References

[1] Y. Kageshima, A. Someno, K. Teshima, K. Domen and H. Nishikiori, *Sustainable Energy Fuels,* 2019, **3**, 2733.
[2] Y. Kageshima, H. Kumagai, T. Minegishi, J. Kubota and K. Domen, *Angew. Chem. Int. Ed.,* 2015, **54**, 7877.
[3] W.-Y. Jeon, Y.-B. Choi, B.-H. Lee, H.-J. Jo, S.-Y. Jeon, C.-J. Lee and H.-H. Kim, *Adv. Mater. Lett.,* 2018, **9**, 220.
[4] B. L. Anderson, A. G. Maher, M. Nava, N. Lopez, C. C. Cummins and D. G. Nocera, *J. Phys. Chem. B,* 2015, **119**, 7422.
[5] T. Minegishi, N. Nishimura, J. Kubota and K. Domen, *Chem. Sci.,* 2013, **4**, 1120.
[6] Y. Kageshima, S. Shiga, H. Kumagai, K. Teshima, K. Domen and H. Nishikiori, *Bull. Chem. Soc. Jpn.,* 2020, **93**, 942.
[7] S. Treimer, A. Tang and D. C. Johnson, *Electroanalysis,* 2002, **14**, 165.
[8] N. Elgrishi, K. J. Rountree, B. D. McCarthy, E. S. Rountree, T. T. Eisenhart and J. L. Dempsey, *J. Chem. Educ.,* 2018, **95**, 2, 197.
[9] M. Maestri, N. Armaroli, V. Balzani, E. C. Constable and A. M. W. C. Thompson, *Inorg. Chem.,* 1995, **34**, 2759.
[10] S. F. McClanahan, R. F. Dallinger, F. J. Holler and J. R. Kincaid, *J. Am. Chem. Soc.,* 1985, **107**, 4853.
[11] M. K. Nazeeruddin, S. M. Zakeeruddin and K. Kalyanasundaram, *J. Phys. Chem.,* 1993, **97**, 9607.
[12] W. H. Quayle and J. H. Lunsford, *Inorg. Chem.,* 1982, **21**, 97.
